# Base- and Additive-Free Carbon Dioxide Hydroboration to Methoxyboranes Catalyzed by Non-Pincer-Type Mn(I) Complexes

**DOI:** 10.1021/acscatal.3c00020

**Published:** 2023-03-31

**Authors:** Sylwia Kostera, Stefan Weber, Ines Blaha, Maurizio Peruzzini, Karl Kirchner, Luca Gonsalvi

**Affiliations:** † Consiglio Nazionale delle Ricerche (CNR), Istituto di Chimica dei Composti Organometallici (ICCOM), Via Madonna del Piano 10, 50019 Sesto Fiorentino, Firenze, Italy; ‡ Institute of Applied Synthetic Chemistry, Vienna University of Technology, Getreidemarkt 9/163-AC, A-1060 Wien, Austria

**Keywords:** carbon dioxide utilization, manganese, organometallics, catalytic hydroboration, methanol

## Abstract

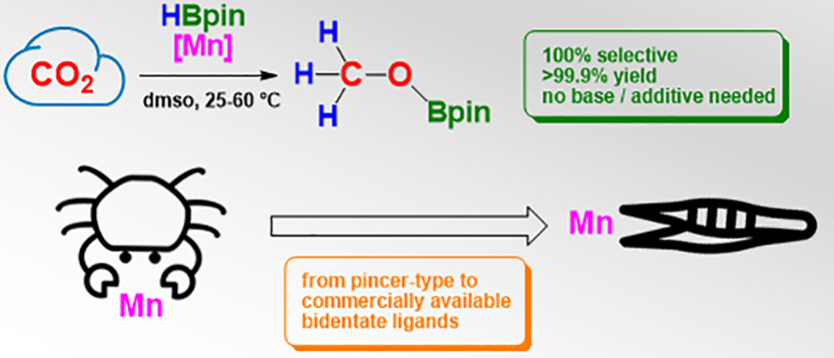

Well-defined, bench stable Mn(I) non-pincer-type complexes were tested as earth-abundant transition metal catalysts for the selective reduction of CO_2_ to boryl-protected MeOH in the presence of pinacolborane (HBpin). Essentially, quantitative yields were obtained under mild reaction conditions (1 bar CO_2_, 60 °C), without the need of any base or additives, in the presence of the alkylcarbonyl Mn(I) bis(phosphine) complexes *fac*-[Mn(CH_2_CH_2_CH_3_)(dippe)(CO)_3_] [**Mn1**, dippe = 1,2-bis(diisopropylphosphino)ethane] and [Mn(dippe)(CO)_2_{(μ-H)_2_(Bpin)}] (**Mn4**), that is obtained by reaction of the bench-stable precatalyst **Mn1** with HBpin via elimination of butanal. Preliminary mechanistic details were obtained by a combination of NMR experiments and monitoring of the catalytic reactions.

Carbon dioxide (CO_2_) represents one of the principal greenhouse gases that are causing environmental concern for their increasing concentration in the atmosphere due to the growing consumption of fossil fuels. In spite of that, the unique characteristics of CO_2_ as an inexpensive, nontoxic, and nonflammable chemical, together with its abundance as raw material, make it attractive for chemists interested in the synthesis of C1-products from renewable sources.^1^ Chemical utilization of CO_2_ is indeed growing in interest, and an increasing amount of literature describing transition metal or metal-free catalytic processes to reduce CO_2_ to useful bulk chemicals such as formic acid (HCOOH) and methanol (CH_3_OH) have been reported, especially in the last two decades.^2^

CH_3_OH, currently produced in about 98 Mt/year quantity worldwide from fossil-based, nonrenewable feedstock under harsh conditions, is a valuable chemical product finding various large-scale direct application.^3^ In the pharmaceutical industry, it is employed as a starting compound for the production of various intermediates. It is also used to obtain formalin, i.e., a saturated aqueous solution of formaldehyde, which has strong bactericidal properties. It is an important solvent in organic synthesis, as a fuel or fuel component in internal combustion engines and as an additive to gasoline to improve the octane number, in household chemicals as a component of washing liquids and antifreeze, and in construction chemicals for the production of adhesives, paints, and varnishes. It is also an antibacterial, bactericidal, and fungicidal substance and utilized in the production of cosmetics and as a contaminant in some goods to deter consumption and inhalation.^4^ Due to its importance, the synthesis of CH_3_OH from renewable feedstock such as CO_2_ would be highly desirable, but efficient catalysts must be designed to decrease the energy demand for the activation of such an inert small molecule by a 6-electron reduction process. Reduction of CO_2_ to methanol or methoxy derivatives can be carried out under homogeneous catalytic conditions in the presence of non-noble^5^ and noble metals complexes^6^ and organocatalysts.^7^

In CO_2_ reduction protocols, hydrogen (H_2_) gas is generally preferred, in particular when it is obtained from sustainable, fossil-free sources and processes (i.e., water electrolysis, water photosplitting). However, the use of a pressurized, explosive gas such as H_2_ has safety drawbacks and high costs of production, storage, and transportation. Alternative reducing agents for CO_2_ such as hydroboranes can be promising to replace H_2_ in the presence of highly active and selective catalysts that bring about the process under atmospheric pressure and mild temperature conditions.^4−7^ Boron-based compounds are generally Lewis acidic and oxophilic enough to enable activation of the C=O bond of carbon dioxide. Reduction of CO_2_ by a hydroborane (Scheme 1) can lead to a variety of products such as formoxyborane (**A**), bis(boryl)acetal (**B**), methoxyborane (**C**), and bis(boryl)ether (**D**).^8^ Therefore, high process selectivity is desired to achieve good atom-efficiency and to avoid distillation to separate the products present in the reaction mixtures.

**Scheme 1:**
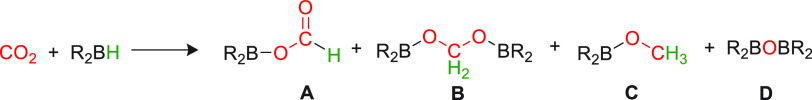
Full Product Range Expected in CO_2_ Hydroboration

Traditionally, platinum group metals have found use as catalysts for CO_2_ hydroboration.^8,9^ In the past decade, part of the chemistry community has focused on the quest for cheaper catalysts based on earth-abundant metals to increase process sustainability.^8^ In particular, manganese, being the third most abundant metal in the Earth’s crust, is receiving a lot of attention in homogeneous catalysis, including CO_2_ reduction processes.^10^ To the best of our knowledge, very few reports are available to date describing efficient manganese catalysts for CO_2_ hydroboration (Chart 1).

**Chart 1:**
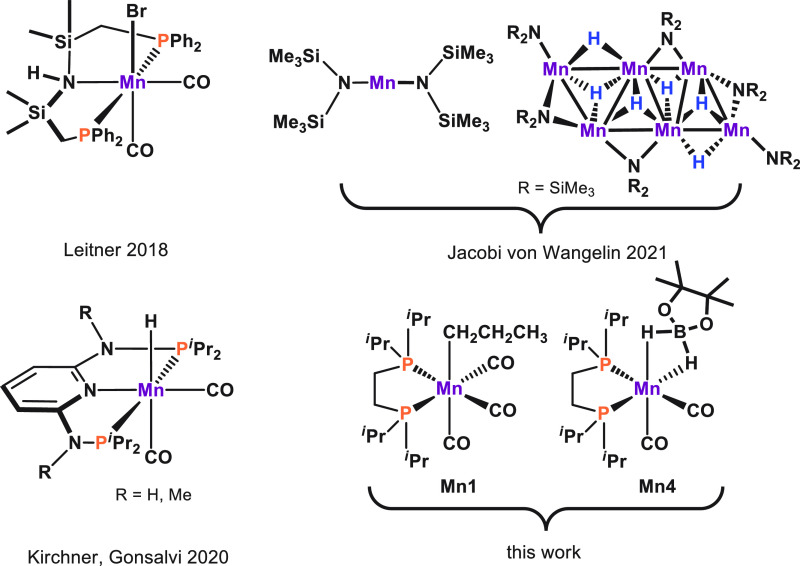
Mn(I) Complexes Used as Catalysts for CO_2_ Hydroboration

Leitner and co-workers described in 2018 the first example of such use. In the presence of complex [MnBr{(Ph_2_PCH_2_SiMe_2_)_2_NH}(CO)_2_], the selective formation of methoxyborane was obtained with high turnover numbers (TON = 883) using pinacolborane (HBpin, 2.76 mmol), CO_2_ (1 atm), catalyst (0.036 mol %), and base (NaO^t^Bu, 0.2 mol %), under solventless conditions at moderate temperature (100 °C).^11^ The reaction mechanism was later studied by other authors by DFT calculations.^12^ We recently described the reduction of CO_2_ to methoxyboranes catalyzed by the well-defined manganese complexes [MnH(PNP^NR^-^i^Pr)(CO)_2_] (R = H or Me) using HBpin and 9-BBN (9-BBN = 9-borabicyclo[3.3.1]nonane) and borates as Lewis acids additives. Good yields in CH_3_OBpin (up to 78%) were obtained after 24 h in THF-*d*_8_ under milder reaction conditions (1 bar CO_2_, 60 °C, 0.224 mmol HBpin, 1 mol % catalyst) and B(OPh)_3_ as cocatalyst (2.24 × 10^–2^ mmol).^13^ Finally, Ghosh and Jacobi von Wangelin reported the hydroboration of CO_2_ (ca. 1 bar) at 80 °C with 100% selectivity to methoxyborane using HBpin and catalysts [Mn(hmds)_2_] and [MnH(hmds)]_6_ (hmds = hexamethyldisilazane), albeit reaching rather low yields (21% and 18% after 72 h, respectively) using 5 mol % of catalysts.^14^

In the past few years, the chemistry and use of a class of textbook, bench-stable, non-pincer-type Mn(I) alkylcarbonyl complexes stabilized by simple and commercially available bis(phosphine) ligands was revisited,^15^ showing that they can work as efficient catalysts for hydrogenation,^16^ hydrosilylation,^17^ and hydroboration processes.^18^ In this class of complexes, precatalyst activation generally occurs by forcing migratory insertion of an alkyl ligand into the Mn–CO bond, with elimination of aldehyde. In hydrogenation processes, for example, this step generates a Mn–H bond and frees one coordination site at the metal, which is available for substrate coordination and the following inner-sphere catalytic mechanism of activation and product release.^15,16^ Recently, we jointly demonstrated that complex *fac*-[Mn(CH_2_CH_2_CH_3_)(dippe)(CO)_3_] [**Mn1**, dippe = 1,2-bis(diisopropylphosphino)ethane] promotes CO_2_ catalytic hydrogenation to formate under mild reaction conditions (75 bar CO_2_/H_2_ total pressure, 80 °C) in the presence of base and Li salts as Lewis acid cocatalysts.^19^

Hereby, we present results on the use of **Mn1** and analogues (Chart 2) as catalysts for the selective hydroboration of CO_2_ to boryl-protected MeOH in the presence of HBpin. Notably, some of these systems gave high yields in the desired product without the need of base or additives, working under mild reaction conditions.

**Chart 2:**
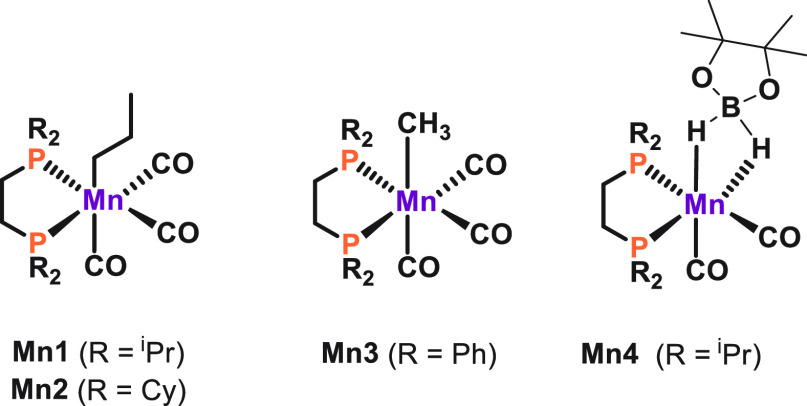
Chemical Drawings of Complexes **Mn1–4**

## Results and Discussion

### Catalytic Tests

The catalytic tests were carried out on NMR tube scale, using catalysts **Mn1–4** (Chart 2), CO_2_ (1 bar), HBpin (0.224 mmol), mesitylene as internal standard (0.056 mmol), no base or additives, monitoring the increase of the yields of C1-products (OCHO)Bpin (**A**), generally observed in small amounts or traces, and (CH_3_O)Bpin (**C**) as the main product (Scheme 1, R_2_B = Bpin) over time by ^1^H NMR spectroscopy (see Experimental Section). Additional ^11^B and ^31^P{^1^H} NMR spectra were collected to obtain more details on the nature of the products and the changes in the catalyst in the course of the reactions. In all tests, the presence of (Bpin)_2_O (**D**) was observed by ^11^B NMR at 22.4 ppm, as expected for stoichiometry reasons. On the other hand, the formation of (Bpin)OCH_2_O(Bpin) (**B**) was never observed in the corresponding ^1^H NMR spectra. The effects of solvent, temperature, and ligands were screened in separate sets of experiments, and the results are hereby described.

**Figure 1:**
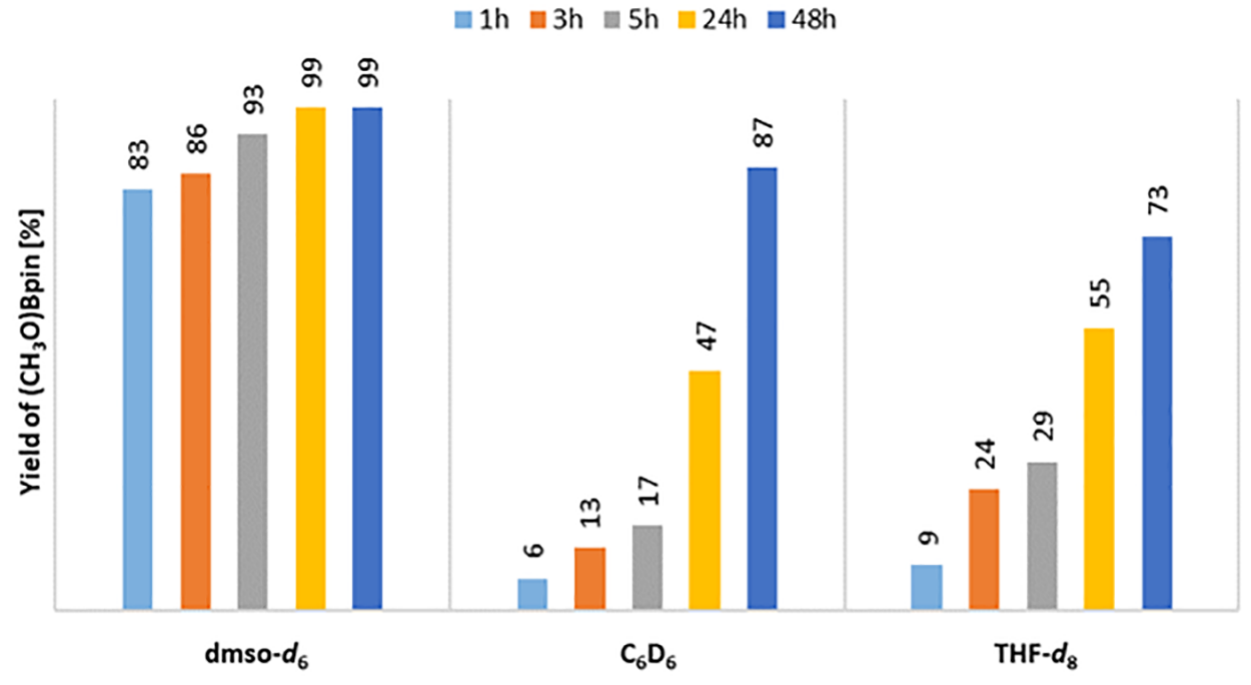
Solvent effect for CO_2_ hydroboration to (CH_3_O)Bpin in the presence of **Mn1**. Values correspond to yields of (CH_3_O)Bpin (mol %). Reaction conditions are detailed in Table S1.

Initially, the activity of precatalyst **Mn1** (1.0 mol % with respect to HBpin) in solvents such as dmso-*d*_6_, THF-*d*_8_, and C_6_D_6_ was studied under standard reaction conditions (60 °C, 1 bar CO_2_, HBpin 0.224 mmol). Noteworthy, dimethyl sulfoxide (dmso) was previously demonstrated to assist CO_2_ activation in hydrogenation^2d^ and in hydrosilylation reactions.^5d^ Under these conditions and using dmso-*d*_6_ as solvent, the reaction gave (CH_3_O)Bpin, identified by the characteristic ^1^H NMR signal at 3.48 ppm and the ^11^B NMR broad signal at 20.9 ppm, as the main C1-product in very high yield (83%) after 1 h, and it increased further after 24 h (99%). Formoxyborane was observed to form in small amounts (ca. 6%) at the beginning of the reaction. No additional increase in yield was observed after 48 h (see Figure 1). Under the same reaction conditions, by changing solvent from dmso-*d*_6_ to either THF-*d*_8_ or C_6_D_6_, (CH_3_O)Bpin formed in yields lower than 60% within 24 h. After 48 h, (CH_3_O)Bpin yields reached 87% and 73% in THF-*d*_8_ and C_6_D_6_, respectively. The results are summarized as column graphs in Figure 1. Reaction conditions and details can be found in Table S1.

**Figure 2:**
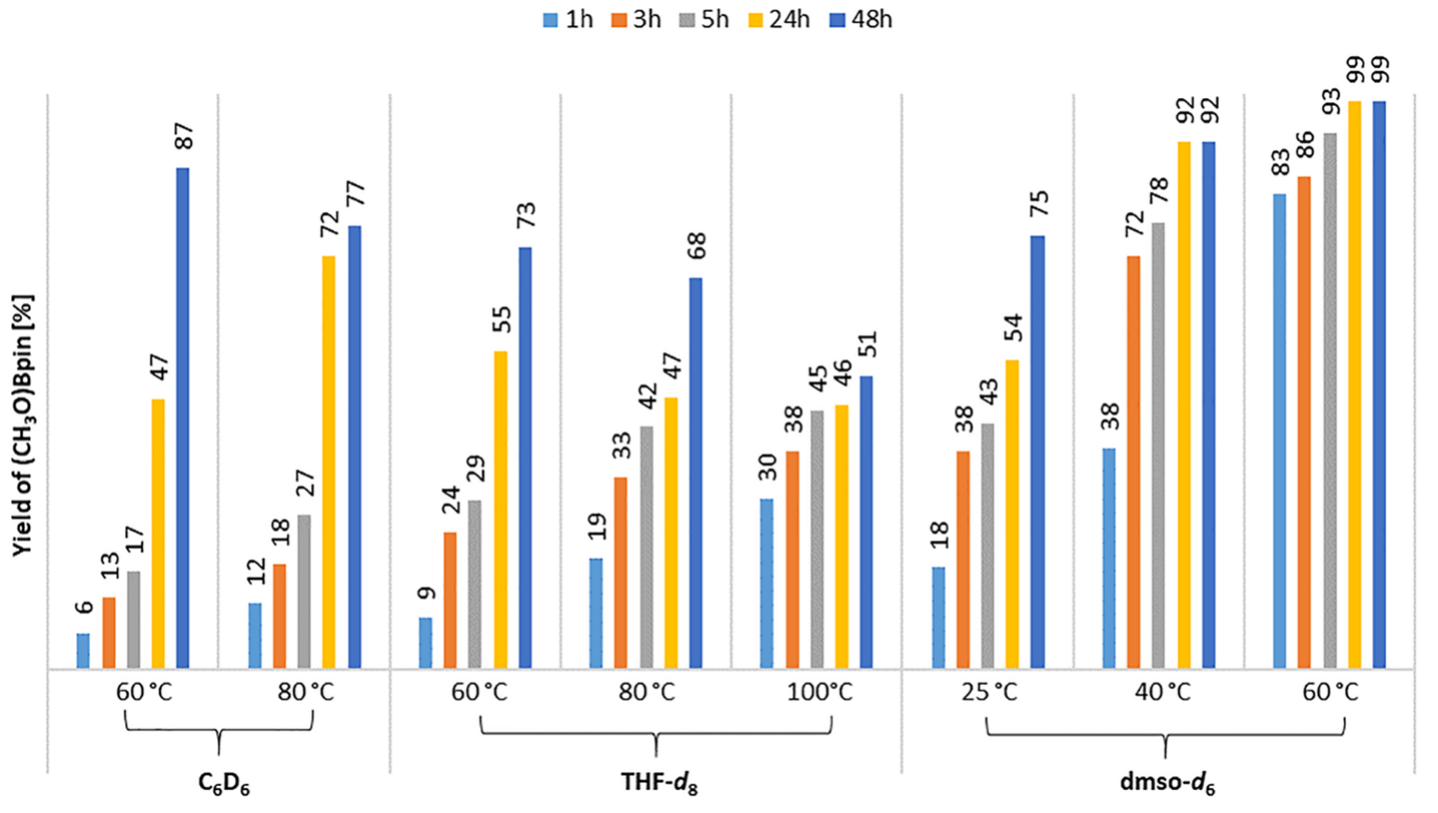
Temperature effect for CO_2_ hydroboration to (CH_3_O)Bpin in the presence of **Mn1**. Values correspond to yields of (CH_3_O)Bpin (mol %). Reaction conditions are detailed in Table S2.

The effect of temperature was then investigated using **Mn1** as catalyst in the three different solvents mentioned above (Figure 2 and Table S2). The best results were confirmed in dmso-*d*_6_ at 60 °C with the formation of (CH_3_O)Bpin in 83% (1 h) and 99% (24 h) yields. By decreasing the temperature to 40 °C, (CH_3_O)Bpin was still obtained in high yield (92%) after 24 h. At room temperature, (CH_3_O)Bpin formation reached 54% yield (24 h) and a remarkable 75% yield after 48 h. In C_6_D_6_ and THF-*d*_8_, the lower performances already observed at 60 °C (Figure 1) were confirmed at 80 °C (max. yield 77% and 68%, 48 h, respectively) and at 100 °C in THF-*d*_8_ (51%, 48 h).

**Figure 3:**
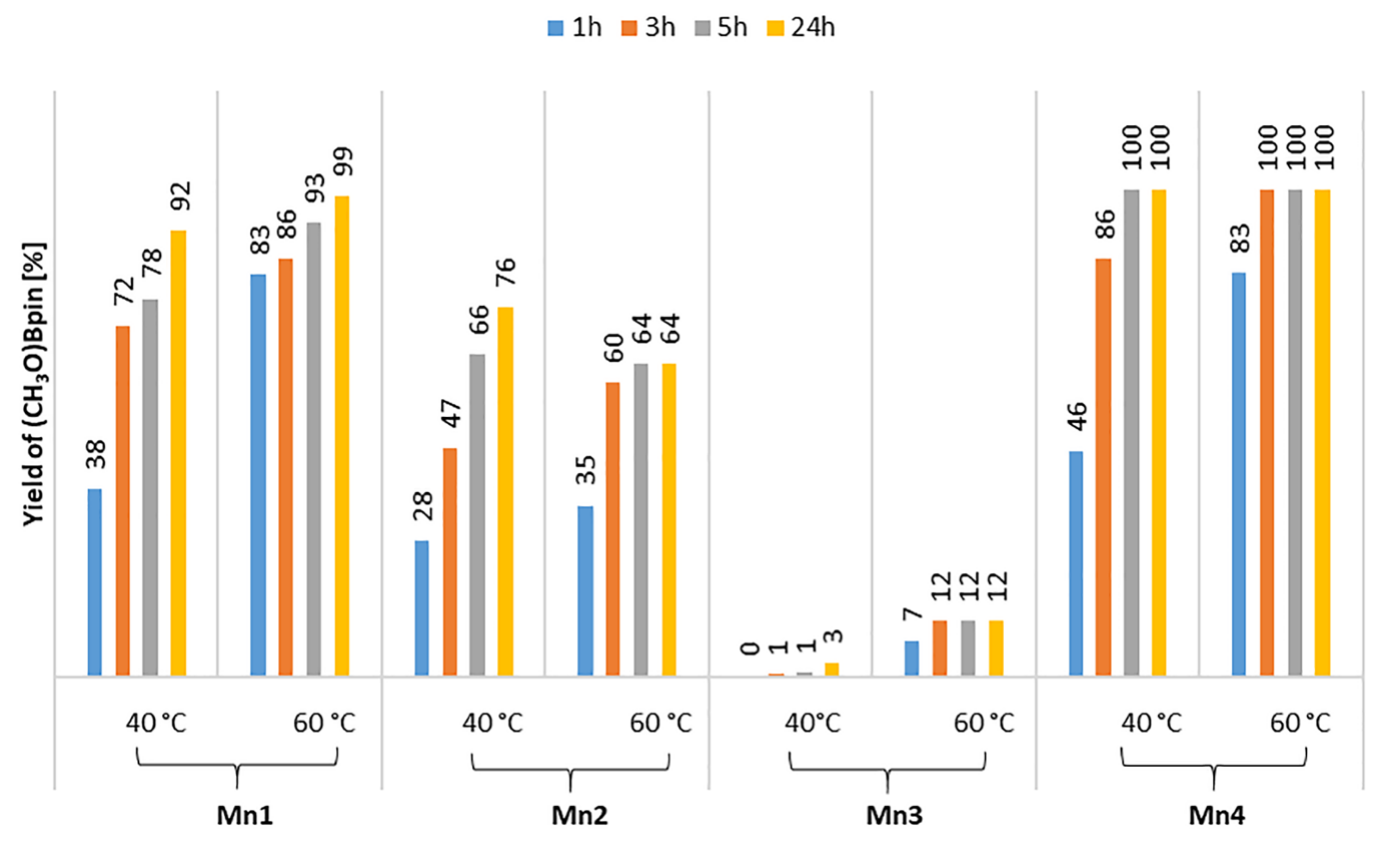
Ligand and temperature effects for CO_2_ hydroboration to (CH_3_O)Bpin in the presence of **Mn1–4**. Values correspond to yields of (CH_3_O)Bpin (mol %). Reaction conditions are detailed in Table S3.

Next, the effect of ligands was tested at 40 and 60 °C using **Mn1** analogues such as **Mn2**,^16^**Mn3**,^20^ and **Mn4**(18) (Chart 2). The results are summarized in Figure 3 (see also Table S3). Compared to **Mn1**, in **Mn2**, the R substituents on P atoms were changed from ^i^Pr to Cy, thus varying the steric hindrance of the complex. In **Mn3**, dippe was replaced by dppe [dppe = 1,2-bis(diphenylphosphino)ethane], and the alkyl ligand was changed from ^n^Pr to CH_3_. Finally, **Mn4** was obtained as previously shown^18^ from the reaction of **Mn1** with HBpin, giving a complex with a (μ-H)_2_(Bpin) ligand that is more labile than ^n^Pr under catalytic conditions.^18^ In other words, **Mn4** may represent an activated form of **Mn1** in hydroboration reactions.

Complex **Mn3** gave poor activity both at 40 and 60 °C with product **C** formation in only 3% and 12% yields after 24 h, respectively, thus confirming that under the conditions applied for CO_2_ hydroboration, CH_3_ is a worse leaving group than ^n^Pr, as previously observed in other catalytic reactions.^15^ In the presence of **Mn2**, lower yields of product **C** were observed after 24 h compared to **Mn1**, both at 40 and 60 °C (76% and 64%, respectively). This is likely related to the higher solubility of **Mn1** than that of **Mn2** in dmso-*d*_6_. Interestingly, **Mn1** and **Mn4** showed a comparable behavior after 1 h of reaction, giving essentially the same yields of **C** at 60 °C. To our delight, almost quantitative yields of (CH_3_O)Bpin were obtained in the presence of **Mn4** after only 3 h at 60 °C or after 5 h at 40 °C. Under the same conditions and time, **Mn1** gave instead 78% yield. Next, another test was run with **Mn4** as catalyst at 25 °C, reaching a yield of (CH_3_O)Bpin of 52% after 5 h and 59% after 24 h.

**Figure 4:**
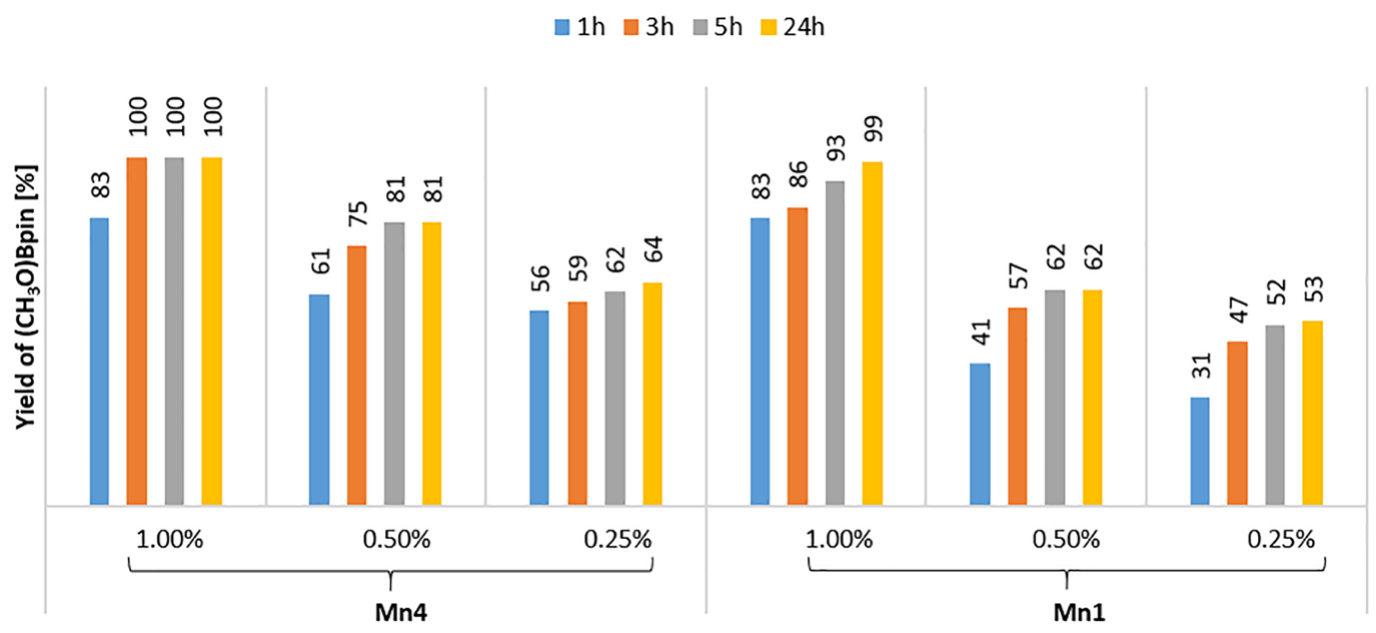
Catalyst amount screening in CO_2_ hydroboration to (CH_3_O)Bpin in the presence of **Mn4** and **Mn1**. Values correspond to yields of (CH_3_O)Bpin (mol %). Reaction conditions are detailed in Table S4.

Finally, the effect of catalyst amount was examined for **Mn1** and **Mn4**, using 0.25, 0.5, 1.0 mol % with respect to HBpin, running the tests in dmso-*d*_6_ at 60 °C for 24 h (Figure 4 and Table S4). At all chosen catalyst to substrate ratios, CO_2_ hydroboration gave (CH_3_O)Bpin as the main C1-product, with final yields varying in the range from 62 to >99%. Although 1.0 mol % was confirmed as the most suitable catalyst amount to obtain the highest yields in (CH_3_O)Bpin, good results were also observed with 0.5 mol % in the case of **Mn4** (81% yield after 5 h). Catalyst deactivation may be responsible for the lower yields observed using 0.5 and 0.25 mol % of **Mn1** and **Mn4** (Figure 4). As recently highlighted in an excellent review article,^21^ a drop in yields may occur when the rate of catalyst deactivation competes with the catalytic rate, especially at high temperatures and at low catalyst concentrations.

A scaled-up (10-fold) Schlenk tube experiment, adapting the reaction conditions used for the highest yielding NMR test (i.e., **Mn1**, 60 °C, 24 h, dmso-*d*_6_), was finally carried out. At the end of the reaction, (CH_3_O)Bpin was obtained in >99.9% yield (see Experimental Section).

### Mechanistic Studies

**Figure 5:**
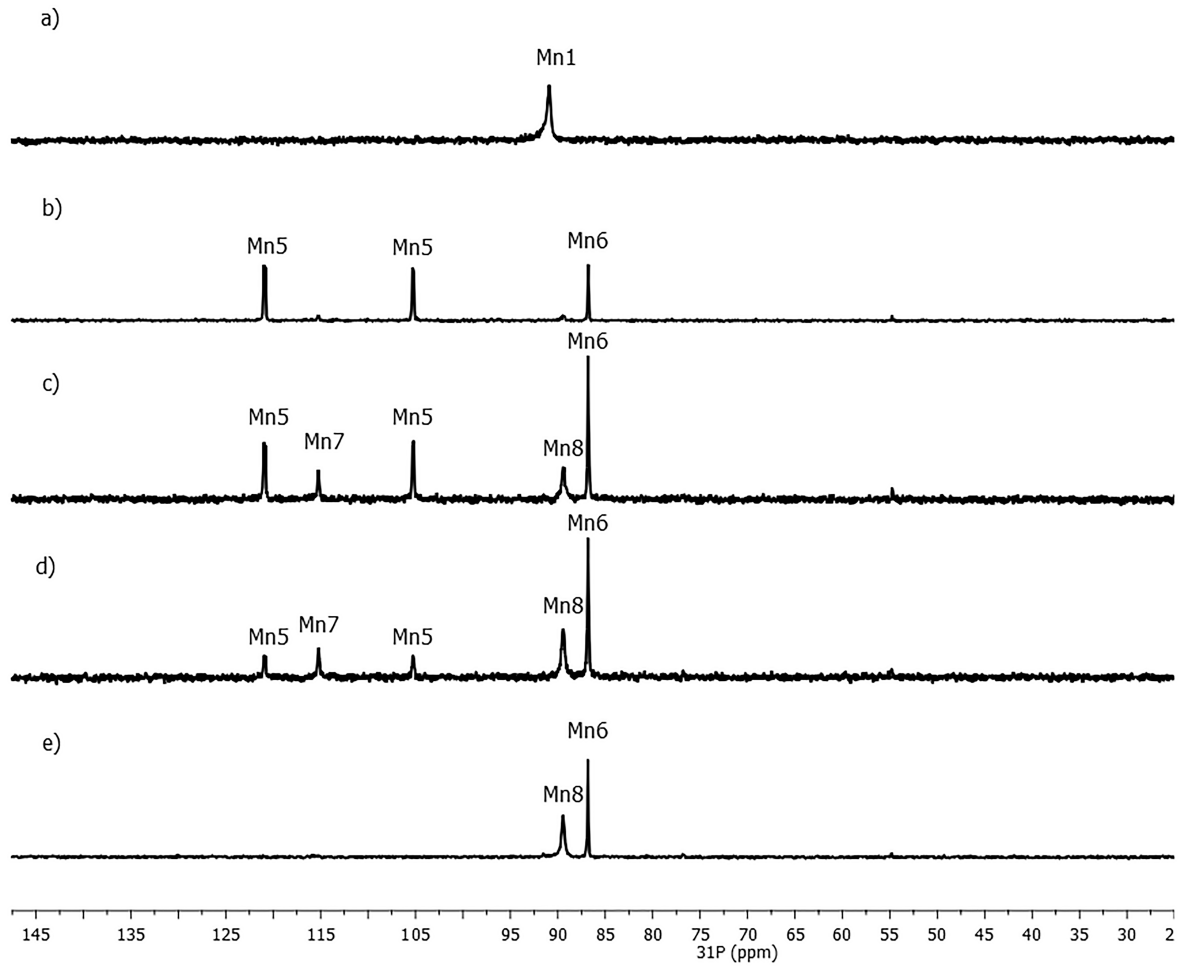
^31^P{^1^H} NMR monitoring of a typical catalytic run. Reaction conditions: **Mn1** (2.24 × 10^–3^ mmol), HBpin (0.224 mmol), CO_2_ (1 bar), 40 °C, dmso-*d*_6_ (0.4 mL), mesitylene as internal standard (0.056 mmol). **Mn1** before (a) reaction and after (b) 1 h; (c) 3 h; (d) 5 h; (e) 24 h.

In order to get insights in the reaction mechanism, ^1^H- and ^31^P{^1^H} NMR spectra were periodically recorded during the course of the catalytic reaction. The ^31^P{^1^H} NMR monitoring of the typical catalytic run in dmso-*d*_6_ is shown in Figure 5. After 1 h, **Mn1** was fully consumed, giving rise to the two new species **Mn5** and **Mn6**. After 3 h, signals due to the new species **Mn7** and **Mn8** were also detected along with **Mn5** and **Mn6**. After 24 h, **Mn6** and **Mn8** were the only species observable in the ^31^P{^1^H} NMR spectrum. Essentially, the same results were achieved employing **Mn4** as precatalyst instead of **Mn1**.

**Chart 3:**
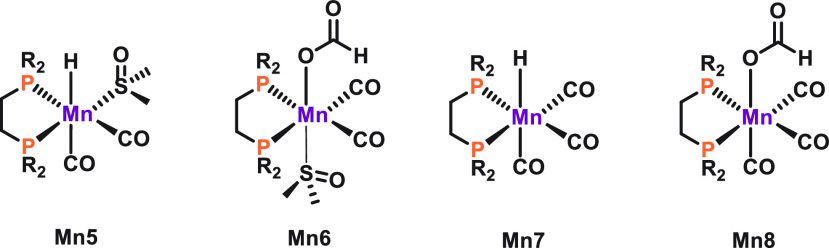
Chemical Drawings of Complexes **Mn5–8**

In order to clarify the nature of the observed new species, additional NMR experiments were carried out. Complex **Mn5** was observed, further than during the catalytic run monitoring, also as the product of the reaction of **Mn1** with 5 equiv of HBpin in dmso-*d*_6_ at 40 °C. It was then synthesized by the reaction of **Mn1** with HBpin (2 equiv) and dmso (2 equiv) in THF at 60 °C (see Experimental Section). In the ^31^P{^1^H} NMR spectrum, **Mn5** is characterized by two doublets at 120.9 and 105.2 ppm, at the same chemical shift values observed during catalytic run monitoring, with a coupling constant ^2^*J*_PP_ = 21.5 Hz. In the corresponding ^1^H NMR spectrum, a doublet of doublets was observed at −6.90 and −7.02 ppm, appearing as a pseudotriplet, with corresponding coupling constants ^2^*J*_HP_ = 49.2 Hz and ^2^*J*_HP_ = 49.3 Hz. Based on these data and on additional ^1^H/^31^P HMBC NMR spectra, we assign the structure of **Mn5** as *cis*-[MnH(dippe)(CO)_2_(κ-*S*-dmso)]. **Mn5** could also be generated upon heating a solution of **Mn4** in dmso-*d*_6_ to 40 °C. This behavior suggests that **Mn4** is likely to be an intermediate in the transformation from **Mn1** to **Mn5** in dmso-*d*_6_. An additional catalytic run under standard conditions (1.0 mol % to HBpin, 60 °C, 24 h, dmso-*d*_6_) in the presence of **Mn5** as catalyst showed selective formation of (CH_3_O)Bpin in >99.9% yield (Figure S.15). Complex **Mn6** was observed in the ^31^P{^1^H} NMR monitoring (Figure 5b) as a singlet at 86.8 ppm, with a broad signal at 7.92 ppm in the corresponding ^1^H NMR spectrum, at a chemical shift value expected for a Mn-formato species (Figure S18). Thus, in the absence of more conclusive evidence, we tentatively assign the structure of **Mn6** as *trans*-[Mn(κ-*O*-OCHO)(dippe)(CO)_2_(dmso)]. Next, the transient species **Mn7**, observed at 115.2 ppm, was identified as the known complex *fac*-[MnH(dippe)(CO)_3_] by comparison with literature data^22^ and independent synthesis in our laboratories (see the Supporting Information). **Mn8** was observed as a broad signal at 89.5 ppm in the ^31^P{^1^H} NMR monitoring (Figure 5c). It was independently obtained by treating **Mn1** with an excess of formic acid (see the Supporting Information). In the ^1^H NMR spectrum (Figure S.24), a sharp signal at 8.17 ppm was observed, at a chemical shift value expected for Mn-OC*H*O moieties. Based on these data, we assign the formula *fac*-[Mn(κ-*O*-OCHO)(dippe)(CO)_3_] to **Mn8**. The chemical drawings of complexes **Mn5–8** are shown in Chart 3.

**Scheme 2:**
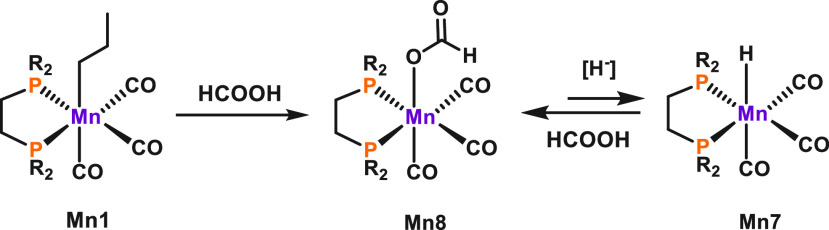
Proposed Side Reactions Leading to **Mn8** and **Mn7** from **Mn1**

Further tests showed that **Mn7** does not react with CO_2_ (1 bar) even if heated to 60 °C for several days. On the other hand, in a separate test, the reaction of **Mn8** with HBpin (excess) produced an unknown boron compound and **Mn7** (Figure S.28). Based on these observations, we suggest that **Mn8** generates from the reaction of **Mn1** with HCOOH formed *in situ* at the beginning of the catalytic run.^13^ Next, the interaction of **Mn8** with a hydride source would give **Mn7**, as shown in Scheme 2. In the presence of even small quantities of HCOOH in solution, **Mn7** may revert to the more thermodynamically stable **Mn8**.

Similarly, **Mn6** may form upon reaction of **Mn5** with HCOOH. In fact, the addition of CO_2_ (1 atm) to a NMR tube containing **Mn5** in dmso-*d*_6_ gave no reaction even after prolonged heating to 60 °C. The two Mn-formato complexes **Mn6** and **Mn8** thus represent resting states of the catalyst, as observed by ^31^P{^1^H} NMR monitoring at the end of the catalytic run (Figure 5e).

Catalyst-free reduction of organic acids to the corresponding alcohols using HBpin was recently reported to occur via facile deoxygenative hydroboration of carboxylic acids to boronate esters and following hydrolysis.^23^ In the framework of the present study, it was therefore important to establish whether the catalyst plays a role in further reduction of HCOOH. To assess this, a NMR tube was charged with dmso-*d*_6_, HCOOH (0.265 mmol), and HBpin at different stoichiometric ratios (1:1; 1:2; 1:5 to HCOOH) under nitrogen. After heating to 60 °C for 24 h, in all cases, only (OCHO)Bpin (**A**) was observed as product in the absence of catalyst. This experiment was then repeated using a HCOOH/HBpin = 1:1 ratio under otherwise identical conditions, adding **Mn1** (2.65 μmol), this time observing the formation of products **A** and **C** in 1:1 ratio (see the Supporting Information), thus confirming the role of the catalyst in the reduction steps beyond HCOOH. The corresponding ^31^P{^1^H} NMR spectrum (Figure S.33), measured at the end of the reaction, showed also for this set of experiments the signals at 89.5 and 86.8 ppm due to **Mn6** and **Mn8**, respectively, as expected upon formation of stable Mn-formato species as catalyst resting states.

## Conclusions

In summary, we have demonstrated that selected Mn(I) complexes supported by simple, commercially available non-pincer-type ligands such as 1,2-bis(diisopropylphosphino)ethane (dippe) are able to bring about the selective hydroboration of CO_2_ to methoxyborane, representing an example of CO_2_ utilization as C1 building block for chemical synthesis, catalyzed by earth-abundant transition metals. Essentially, quantitative yields (>99.9%) in the desired product were obtained using as low as 1 mol % of catalysts under mild reaction conditions (1 bar CO_2_, 25–60 °C) within 5 to 24 h, depending on the complex used, *in the absence of any base or additive*, representing a significant improvement over known protocols based on Mn.

## Experimental Section

### Typical Procedure for CO_2_ Hydroboration (NMR Tube Scale)

A stock solution containing the catalyst (0.56–2.24 × 10^–3^ mmol), HBpin (0.224 mmol), and mesitylene as internal standard (0.056 mmol) in the desired solvent (0.4 mL) was added under nitrogen to a J-Young NMR tube. Once prepared, the sample was degassed via three freeze–pump–thaw cycles and then CO_2_ (1 bar) was added using a Schlenk line. The NMR tube was then placed in an oil bath kept at the desired temperature (25, 40, 60, 80, or 100 °C) for the set reaction time. The formation of C1-products was monitored throughout the duration of the experiment using ^1^H NMR spectroscopy and quantified by signal integration versus the mesitylene internal standard. Additional ^31^P{^1^H} and ^11^B NMR spectra were also run to obtain further qualitative information. Each test was repeated at least twice to check for reproducibility. The yields of C1-products were obtained with an average error of ca. 6%.

### Typical Procedure for CO_2_ Hydroboration (Schlenk Tube Scale)

The procedure described above was adapted for a Schlenk tube scale test. **Mn1** (2.24 × 10^–2^ mmol) was dissolved in dmso-*d*_6_ (4.0 mL) in a Schlenk tube equipped with a Rotaflo Teflon cap. HBpin (2.24 mmol) and mesitylene as internal standard (0.56 mmol) were then added under nitrogen to the mixture. The solution was frozen using a liquid nitrogen bath; the tube was degassed via three freeze–pump–thaw cycles, and then, CO_2_ (1 bar) was added using a Schlenk line. The tube was warmed to room temperature and then heated to 60 °C for 24 h. At the end of the reaction, the excess CO_2_ was vented and an aliquot of the solution was analyzed by ^1^H NMR. (CH_3_O)Bpin was obtained in >99.9% yield as determined by integration of the ^1^H NMR signal at 3.48 ppm against the internal standard. The test was repeated twice to check for reproducibility.

### Synthesis of *fac*-[Mn(CH_2_CH_2_CH_3_)(dcpe)(CO)_3_] (**Mn2**)

This compound was prepared using the precursor [MnBr(dcpe)(CO)_3_] that was synthesized according to the literature.^24^**Mn2** was subsequently prepared in analogous fashion to **Mn1**(16) using [MnBr(dcpe)(CO)_3_] (0.765 g, 1.27 mmol) and propyl bromide (17 mL). Crude **Mn2** was extracted with *n*-pentane (90 mL), and the solvent was removed in vacuo to yield a pale yellow solid (0.359 g, 47%). ^1^H NMR (δ, 400 MHz, C_6_D_6_, 20 °C): 2.29–1.88 (m, 14H), 1.85–1.04 (m, 39H), 0.36 (quin, *J* = 8.5 Hz, 2H). ^13^C{^1^H} NMR (δ, 101 MHz, C_6_D_6_, 20 °C): 227.9 (CO), 220.5 (CO), 40.2 (vt, *J* = 10.1 Hz), 35.0 (vt, *J* = 7.0 Hz), 31.3 (vt, *J* = 3.8 Hz), 31.1, 30.8, 29.8, 29.2, 28.5–27.9 (m), 27.0, 26.7, 22.5 (vt, *J* = 18.0 Hz), 12.0 (vt, *J* = 14.7 Hz). ^31^P{^1^H} NMR (δ, 162 MHz, C_6_D_6_, 20 °C): 82.9 (s). ATR-IR (solid, cm^–1^): 1969 (ν_CO_), 1895 (ν_CO_), 1855 (ν_CO_). HRMS (TOF ESI^+^): *m*/*z* calculated for C_32_H_55_MnO_3_P_2_ [M – H]^+^: 605.3079, found 605.3098.

### Synthesis of *cis*-[MnH(dippe)(CO)_2_(κ-*S*-dmso)] (**Mn5**)

To a solution of **Mn1** (15 mg, 3.36 × 10^–5^ mmol) in THF (0.5 mL), pinacolborane (8.6 mg, 6.72 × 10^–5^ mmol, 2 equiv) and dmso-*d*_6_ (4.24 mg, 5.04 × 10^–5^ mmol, 1.5 equiv) were added. The solution was stirred at 60 °C for 4 h, monitoring by ^31^P{^1^H} NMR. All volatiles were then removed in vacuo. The obtained white solid was dried in vacuo and analyzed by ^1^H and ^31^P{^1^H} NMR spectroscopy. The complex, highly soluble in most common organic solvents, was obtained with impurities derived from byproducts of pinacolborane redistribution. Yield (crude): 65%. ^1^H NMR (δ, 400 MHz, dmso-*d*_6_, 20 °C): 2.5 (m), 2.23 (m), 2.03 (m), 1.78 (m), 1.63 (m), 1.37 (m), 1.21–1.05 (m), −6.90, −7.02 ppm (dd, ^2^*J*_HP_ = 49.2, ^2^*J*_HP_ = 49.3 Hz, 1H). ^31^P{^1^H} NMR (δ, 162 MHz, dmso-*d*_6_, 20 °C): 123.11 (d, ^2^*J*_PP_ = 21.9 Hz), 107.41 (d, ^2^*J*_PP_ = 22.7 Hz).

## Supporting Information Available

General methods and materials, synthesis and characterization of manganese complexes, general procedures for catalytic tests, tables of catalytic data, additional NMR experiments, and selected NMR spectra (PDF)
