# Supplementary material for: Base- and Additive-Free Carbon Dioxide Hydroboration to Methoxyboranes Catalyzed by Non-Pincer-Type Mn(I) Complexes
Source: ACS Catal. 2023 Mar 31;13(8):5236–44. doi: 10.1021/acscatal.3c00020 (PMC10127281; doi:10.1021/acscatal.3c00020)
Supplement: Supplementary file 1 — cs3c00020_si_001.pdf [file cs3c00020_si_001.pdf]

# Base- and Additive-free Carbon Dioxide Hydroboration to Methoxyboranes Catalyzed by Non-pincer-type Mn(I) complexes.

Sylwia Kostera,[a] Stefan Weber,[b] Ines Blaha,[b] Maurizio Peruzzini,[a] Karl Kirchner*,[b] and Luca Gonsalvi*,[a]

[a] Consiglio Nazionale delle Ricerche (CNR), Istituto di Chimica dei Composti Organometallici (ICCOM), Via Madonna del Piano 10, 50019 Sesto Fiorentino (Firenze), Italy. Email: l.gonsalvi@iccom.cnr.it

[b] Institute of Applied Synthetic Chemistry, Vienna University of Technology, Getreidemarkt 9/163-AC, A-1060 Wien, Austria. Email: karl.kirchner@tuwien.ac.at

# SUPPORTING INFORMATION

1. General methods and materials
2. Synthesis and characterization of Mn complexes
3. General procedures for catalytic tests
4. Tables of catalytic hydroboration results
5. Selected NMR spectra – catalytic runs
6. Selected NMR spectra – mechanistic studies
7. References

## 1. GENERAL METHODS AND MATERIALS

The complexes [Mn($CH_2CH_2CH_3$)(dippe)$(CO)_3$] (**Mn1**), [Mn($CH_3$)(dcpe)$(CO)_3$] (**Mn3**), [Mn(dippe)$(CO)_2$($\mu$-H)$_2$(Bpin)] (**Mn4**) and [MnH(dippe)$(CO)_3$] (**Mn7**) were synthesized as previously described.[1-4] All manipulations were carried out using standard Schlenk techniques under nitrogen atmosphere. Pinacolborane (HBpin) was obtained from commercial suppliers and used as received under nitrogen atmosphere. $CO_2$ (gas cylinder, 7 bar) was purchased from Air Liquide and used as received. Solvents were freshly distilled over appropriate drying agents, collected over Linde type 3Å or 4Å molecular sieves under nitrogen, and degassed with nitrogen or argon. Deuterated solvents for NMR measurements were purchased from commercial suppliers and stored onto activated 4Å molecular sieves under Ar before use. The $^{1}H$, $^{13}C\{^{1}H\}$, and $^{31}P\{^{1}H\}$ NMR spectra were recorded on a Bruker AVANCE-250 spectrometer (operating at 250.13, 101.26, and 62.90 MHz, respectively), on a Bruker Avance II 300 spectrometer (operating at 300.13, 75.47, and 121.50 MHz, respectively) and a Bruker Avance II 400 spectrometer (operating at 400.13, 100.61, and 161.98 MHz, respectively) at room temperature. Peak positions were calibrated against the residual solvent resonance ($^{1}H$) or the deuterated solvent multiplet ($^{13}C$). $^{31}P\{^{1}H\}$ NMR were referenced to 85% $H_3PO_4$, with the downfield shift taken as positive.

## 2. SYNTHESIS AND CHARACTERIZATION OF Mn COMPLEXES

### 2.1.1. [Mn($CH_2CH_2CH_3$)(dippe)$(CO)_3$] (**Mn1**)

The complex was synthesized as previously reported.[1]

$^{1}H$ NMR (δ, 400 MHz, $CD_2Cl_2$, 20 °C): 2.47 – 2.03 (m, 7H), 1.98 – 1.52 (m, 13H), 1.52 – 1.07 (m, 33H), 0.93 (t, J = 7.1 Hz, 2H), 0.10 (quin, J = 17.0 Hz, J = 8.5 Hz, 2H)). $^{13}C\{^{1}H\}$ NMR (δ ,101 MHz, $CD_2Cl_2$, 20 °C): 30.1, 27.9 (vt, J = 9.0 Hz), 24.0 (vt, J = 18.2 Hz), 21.6, 19.8 (vd, J = 10.1 Hz), 19.3, 18.4, 9.5 (vt, J = 15.1 Hz), (CO not observed). $^{31}P\{^{1}H\}$ NMR (δ, 162 MHz, $CD_2Cl_2$, 20 °C): 90.9 (s). ATR-IR (solid, $cm^{-1}$): 1964 (νCO), 1878 (νCO), 1858 (νCO). HRMS (TOF ESI$^+$): m/z calculated for $C_{17}H_{31}MnO_3P_2$ $[M-C_3H_7]^+$: 401.1202, found 401.1200.

### 2.1.2 [Mn($CH_3$)(dppe)$(CO)_3$] (**Mn3**)

The complex was synthesized as previously reported.[2]

$^{1}H$ NMR (δ, 400 MHz, $CDCl_3$): 7.78 (tt, J = 8.0, 1.5 Hz, 4H), 7.50 (tt, J = 7.9, 1.4 Hz, 4H), 7.15 – 6.98 (m, 11H), 2.55 – 2.31 (m, 2H), 2.29 – 1.96 (m, 2H), -0.72 (t, J = 9.8 Hz, 3H) ppm. $^{31}P\{^{1}H\}$ NMR (δ, 162 MHz, $C_6D_6$): 87.6 ppm. ATR-IR ($cm^{-1}$): 1997 (νCO), 1918 (νCO), 1893 (νCO).

2.1.3. [Mn(dippe)(CO)$_2$(μ-H)$_2$(Bpin)] (**Mn4**)

The complex was synthesized as previously reported.[3]

$^1$H NMR (δ, 400 MHz, THF-*d*$_8$): 2.56 (m, 1H), 2.41 – 2.28 (m, 1H), 2.23 (dq, J = 11.0, 7.0 Hz, 1H), 2.06 – 1.89 (m, 3H), 1.34 – 1.15 (m, 19H), 1.13 (d, J = 7.1 Hz, 4H), 1.10 (q, J = 3.2 Hz, 13H), 1.00 (dd, J = 15.2, 7.1 Hz, 3H), -10.5 – -11.2 (m, 1H), -11.8 – -12.7 (m, 1H). $^{13}$C {$^1$H} NMR (δ, 63 MHz, THF-*d*$_8$): 82.4, 29.5 (d, J = 22.7 Hz), 27.8 (dd, J = 21.8, 1.4 Hz), 26.7 (dd, J = 20.6, 2.4 Hz), 24.4 – 24.0 (m), 23.9, 23.6, 21.1, 20.8 (d, J = 3.9 Hz), 20.4 (d, J = 4.2 Hz), 18.7 (d, J = 2.1 Hz), 18.3 (dd, J = 5.5, 3.6 Hz), 18.1 – 17.8 (m), 17.0, 16.6 (d, J = 6.3 Hz). CO not observed $^{31}$P{$^1$H} (δ, 163 MHz, THF-*d*$_8$): 116.3 (br, 1P), 97.3 (br, 1P). ATR-IR (solid, cm$^{-1}$): 1930 (νCO), 1856 (νCO). HRMS (TOF ESI$^+$): m/z calculated for $C_{22}H_{48}BMnO_4P_2Na$ [M$^+$Na]$^+$: 526.2399, found 526.2308.

2.1.4. *fac*-[MnH(dippe)(CO)$_3$] (**Mn7**)

The complex was synthesized as previously reported.[4]

$^1$H NMR (δ, 400 MHz, THF-*d*$_8$): 2.27–2.07 (m, 4H), 1.77–1.59 (*m),* 1.73 (s, THF), 1.28–1.12 (m, 24H), −9.22 (*t*, $^2J_{HP}$ = 48 Hz, 1H). $^{31}$P{$^1$H} NMR (δ, 161.9 MHz, THF-*d*$_8$): 119.16 (*s*). $^{31}$P NMR (δ, 161.9 MHz, THF-*d*$_8$): 119.15 (bs). FTIR (ATR): *ν*C–H (cm$^{-1}$) 2983.49 w, 2960.89 m, 2932.24 w, 2870.27 w; *ν*C–O (cm$^{-1}$) 1971.63 s, 1891.01 s, 1868.05 s. Anal. Calcd for $C_{17}H_{33}MnO_3P_2$: C, 50.75; H, 8.27. Found: C, 50.76; H, 8.58.

2.1.5 *fac*-[Mn(κ-*O*-OCHO)(dippe)(CO)$_3$] (**Mn8**)

HCOOH (0.11 mmol, 5 equiv.) was added to a solution of **Mn1** (0.02 mmol) in THF (500 µL). The solution was stirred at 70 °C for 48 h. All volatiles were removed in vacuo and the remaining solid was washed with *n*-pentane (3 x 0.5 mL). The obtained white solid was dried in vacuo and analyzed by $^1$H and $^{31}$P{$^1$H} NMR spectroscopy. Yield = 22%.

$^1$H NMR (400 MHz, $CD_2Cl_2$, 20 °C) δ 8.17 (s, 1H), 2.47 – 2.21 (m, 4H), 2.08 – 1.83 (m, 4H), 1.58 – 0.98 (m, 24H) ppm. $^{31}$P{$^1$H} NMR (δ, 162 MHz, $CD_2Cl_2$, 20 °C): 88.9 ppm (s).

## 3. GENERAL PROCEDURES FOR CATALYTIC TESTS

### 3.1. NMR TUBE SCALE

A Wildmad quick pressure valve J-Young NMR tube fitted with a Teflon valve (inner volume ca. 3.5 mL) was charged with solvent (0.4 mL), pinacolborane (HBpin, 0.224 mmol), catalyst in the chosen amount, and mesitylene (0.056 mmol) as internal standard. The NMR tube was then connected to a high vacuum line, the solution was frozen with a liquid nitrogen bath, and the headspace was evacuated. The tube was then charged with $CO_2$ (1 bar, ca. 0.14 mmol considering the headspace), defrozen and either quickly transferred to the NMR probe (for tests at 25 °C) or kept in an oil bath set to the desired temperature and then monitored by $^1H$ NMR at chosen intervals of time, depending on the type of experiment. All experiments were repeated at least twice to check for reproducibility (average error ca. 6%).

### 3.2. SCHLENK TUBE SCALE

A Schlenk tube with a Teflon valve cap was charged with solvent (4.0 mL), pinacolborane (2.24 mmol), **Mn1** (2.24 mmol, 11 mg) and mesitylene (0.56 mmol) as internal standard. The Schlenk tube was then connected to a high vacuum line, the solution was frozen with a liquid nitrogen bath, and the headspace was evacuated. The Schlenk tube was then charged with $CO_2$ (1 bar, ca. 1.4 mmol considering the headspace), defrozen and kept in an oil bath set to 60 °C. The reaction was stopped after 24 h and was analyzed by $^1H$ NMR and $^{31}P\{^1H\}$ NMR spectroscopy. The experiment was repeated twice to check for reproducibility (average error ca. 6%).

## 4. TABLES OF CATALYTIC HYDROBORATION RESULTS

**Table S1. Solvent effect for $CO_2$ hydroboration to ($CH_3O$)Bpin in the presence of Mn1.**[a]

| Time (h) | dmso-$d_6$[b] | $C_6D_6$[b] | THF-$d_8$[b] |
|---|---|---|---|
| 1 | 83 | 6 | 9 |
| 3 | 86 | 13 | 24 |
| 5 | 93 | 17 | 29 |
| 24 | 99 | 47 | 55 |
| 48 | 99 | 87 | 73 |

[a] Reaction conditions: **Mn1** ($2.24 \times 10^{-3}$ mmol), HBpin (0.224 mmol), $CO_2$ (1 bar), 60 °C, solvent (0.4 mL). Product yields obtained by $^1H$ NMR signals integration against a mesitylene internal standard (0.056 mmol). All test repeated at least twice. Average error = 6%. [b] Yield of ($CH_3O$)Bpin.

**Table S2. Temperature effect for $CO_2$ hydroboration to $(CH_3O)Bpin$ in the presence of Mn1.**[a]

| Time (h) | $C_6D_6$[b] | | THF-$d_8$[b] | | | dmso-$d_6$[b] | | |
|---|---|---|---|---|---|---|---|---|
| °C | 60 | 80 | 60 | 80 | 100 | 25 | 40 | 60 |
| 1 | 6 | 12 | 9 | 19 | 30 | 18 | 38 | 83 |
| 3 | 13 | 18 | 24 | 33 | 38 | 38 | 72 | 86 |
| 5 | 17 | 27 | 29 | 42 | 45 | 43 | 78 | 93 |
| 24 | 47 | 72 | 55 | 47 | 46 | 54 | 92 | 99 |
| 48 | 87 | 77 | 73 | 68 | 51 | 75 | 92 | 99 |

[a] Reaction conditions: **Mn1** (2.24 x $10^{-3}$ mmol), HBpin (0.224 mmol), $CO_2$ (1 bar), solvent (0.4 mL). Product yields obtained by $^1$H NMR signals integration against a mesitylene internal standard (0.056 mmol). All test repeated at least twice. Average error = 6%. [b] Yield of $(CH_3O)Bpin$.

**Table S3. Ligand and temperature effects for $CO_2$ hydroboration to $(CH_3O)Bpin$ in the presence of Mn1-4.**[a]

| Time (h) | **Mn1**[b] | | **Mn2**[b] | | **Mn3**[b] | | **Mn4**[b] | |
|---|---|---|---|---|---|---|---|---|
| °C | 40 | 60 | 40 | 60 | 40 | 60 | 40 | 60 |
| 1 | 38 | 83 | 28 | 35 | < 1 | 7 | 46 | 83 |
| 3 | 72 | 86 | 47 | 60 | < 1 | 12 | 86 | > 99 |
| 5 | 78 | 93 | 66 | 64 | < 1 | 12 | > 99 | > 99 |
| 24 | 92 | 99 | 76 | 64 | 3 | 12 | > 99 | > 99 |

[a] Reaction conditions: catalyst (2.24 x $10^{-3}$ mmol), HBpin (0.224 mmol), $CO_2$ (1 bar), dmso-$d_6$ (0.4 mL). Product yields obtained by $^1$H NMR signals integration against a mesitylene internal standard (0.056 mmol). All test repeated at least twice. Average error = 6%. [b] Yield of $(CH_3O)Bpin$.

**Table S4. Catalyst amount screening in $CO_2$ hydroboration to $(CH_3O)Bpin$ in the presence of Mn4 and Mn1.**[a]

| Time (h) | **Mn4**[b] | | | **Mn1**[b] | | |
|---|---|---|---|---|---|---|
| Mn [%] | 1 | 0.5 | 0.25 | 1 | 0.5 | 0.25 |
| 1 | 83 | 61 | 56 | 83 | 41 | 31 |
| 3 | > 99 | 75 | 59 | 86 | 57 | 47 |
| 5 | > 99 | 81 | 62 | 93 | 62 | 52 |
| 24 | > 99 | 81 | 64 | 99 | 62 | 53 |

[a] Reaction conditions: catalyst (2.24/ 1.12/ 0.56 x $10^{-3}$ mmol), HBpin (0.224 mmol), $CO_2$ (1 bar), 60 °C, dmso-$d_6$ (0.4 mL). Product yields obtained by $^1$H NMR signals integration against a mesitylene internal standard (0.056 mmol). All test repeated at least twice. Average error = 6%. [b] Yield of $(CH_3O)Bpin$.

## 5. SELECTED NMR SPECTRA - CATALYTIC RUNS

### 5.1. $CO_2$ hydroboration in the presence of Mn1

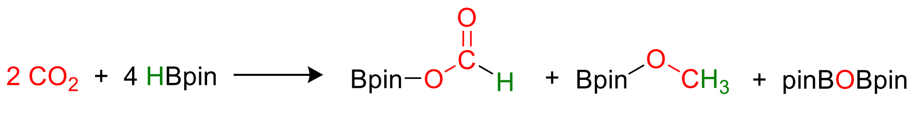


A C D

### 5.1.1. $CO_2$ hydroboration in $C_6D_6$ at 60 °C

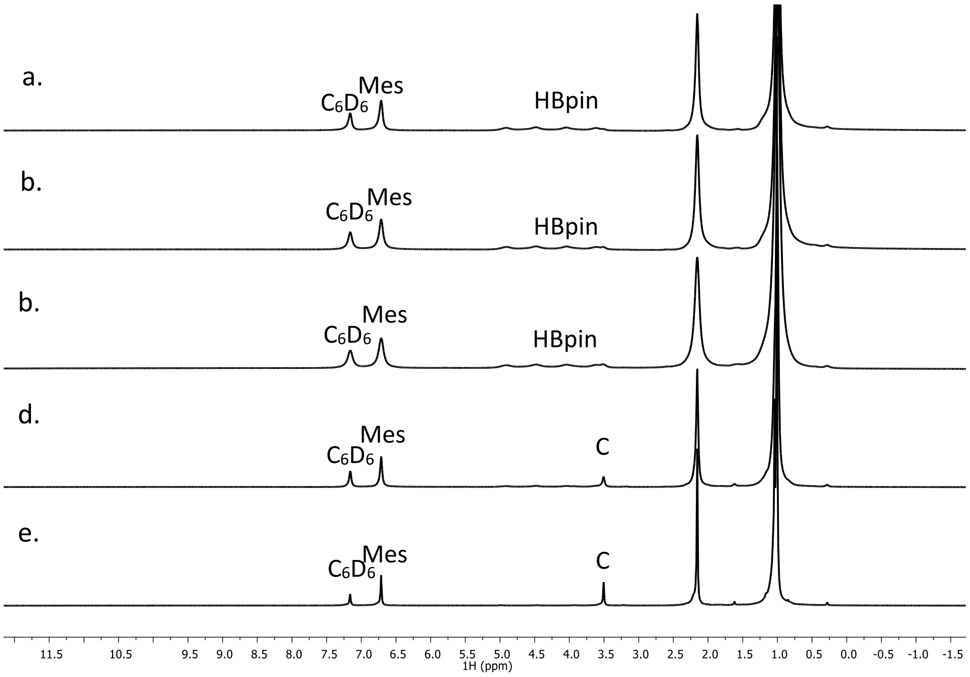


**Figure S.1.** $^{1}H$ NMR reaction monitoring. Reaction conditions: **Mn1** ($2.24 \times 10^{-3}$ mmol), HBpin (0.224 mmol), $CO_2$ (1 bar), 60 °C, $C_6D_6$ (0.4 mL), mesitylene as internal standard (0.056 mmol). Reaction time: a) 1 h; b) 3 h; c) 5 h; d) 24 h; e) 48 h.

### 5.1.2. $CO_2$ hydroboration in $C_6D_6$ at 80 °C

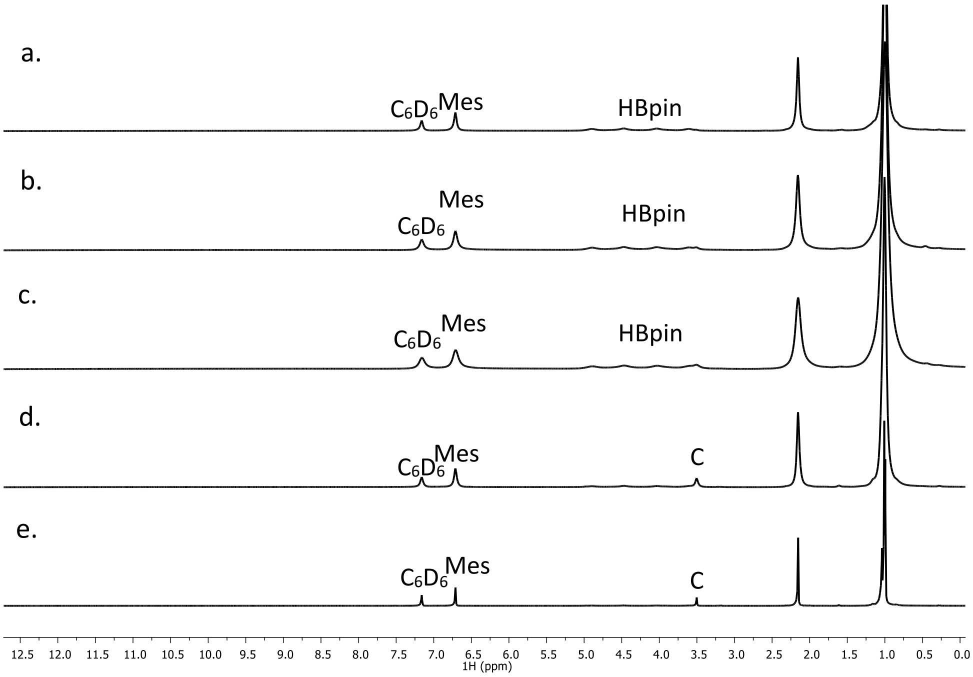


**Figure S.2.** $^{1}H$ NMR reaction monitoring. Reaction conditions: **Mn1** ($2.24 \times 10^{-3}$ mmol), HBpin (0.224 mmol), $CO_2$ (1 bar), 80 °C, $C_6D_6$ (0.4 mL), mesitylene as internal standard (0.056 mmol). Reaction time: a) 1 h; b) 3 h; c) 5 h; d) 24 h; e) 48 h.

## 5.1.3. $CO_2$ hydroboration in THF-d$_8$ at 60 °C

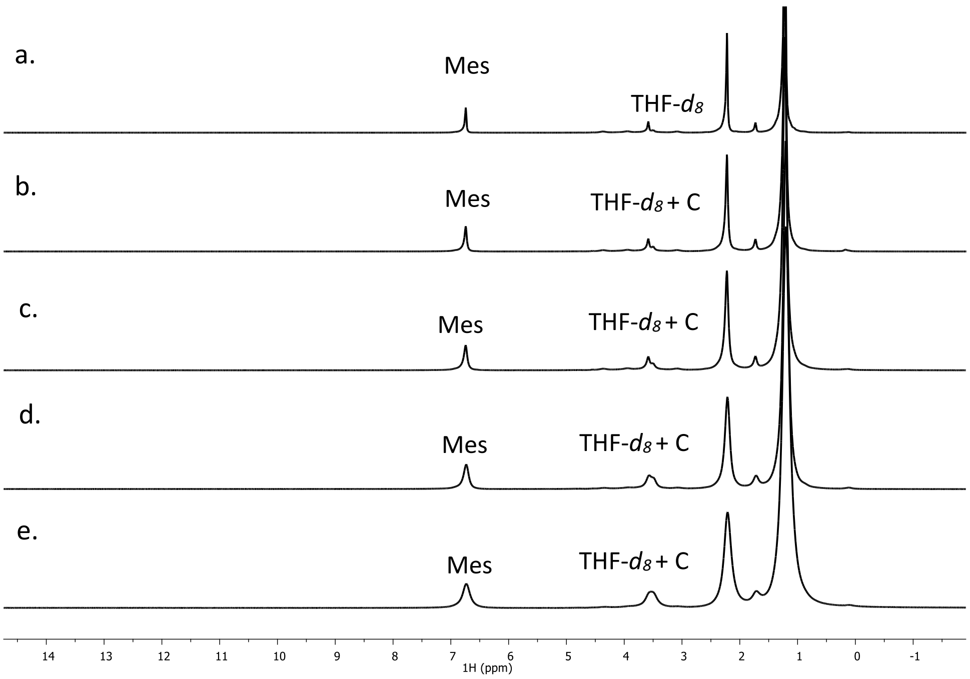


**Figure S.3.** $^1$H NMR reaction monitoring. Reaction conditions: **Mn1** ($2.24 \times 10^{-3}$ mmol), HBpin (0.224 mmol), $CO_2$ (1 bar), 60 °C, THF-*d$_8$* (0.4 mL), mesitylene as internal standard (0.056 mmol). Reaction time: a) 1 h; b) 3 h; c) 5 h; f) 24 h; e) 48 h.

## 5.1.4. $CO_2$ hydroboration in THF-d$_8$ at 80 °C

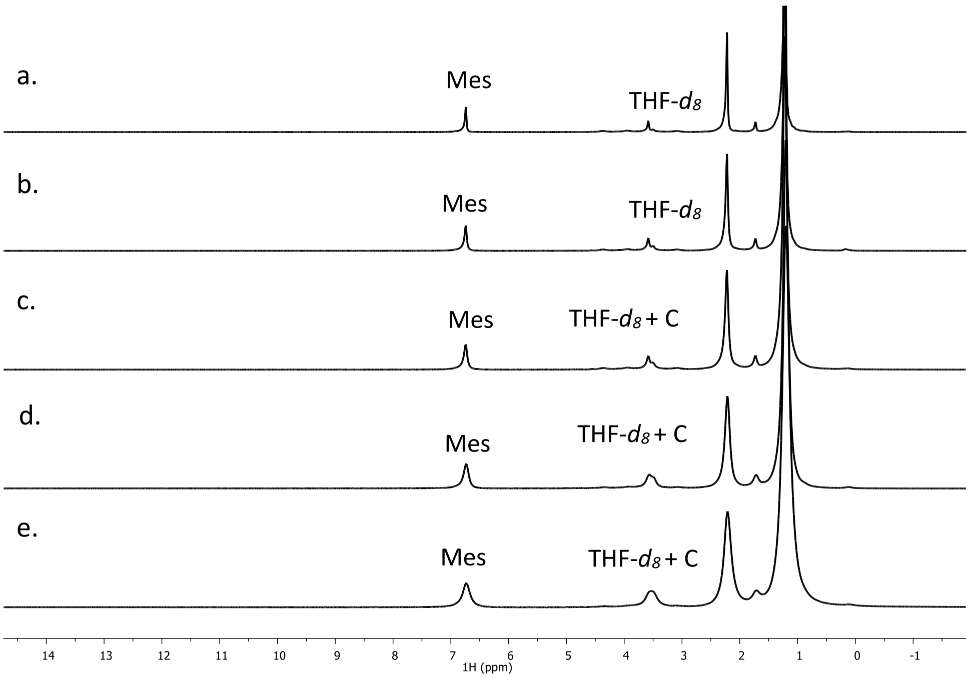


**Figure S.4.** $^1$H NMR reaction monitoring. Reaction conditions: **Mn1** ($2.24 \times 10^{-3}$ mmol), HBpin (0.224 mmol), $CO_2$ (1 bar), 80 °C, THF-*d$_8$* (0.4 mL), mesitylene as internal standard (0.056 mmol). Reaction time: a) 1 h; b) 3 h; c) 5 h; d) 24 h; e) 48 h.

## 5.1.5. $CO_2$ hydroboration in THF-d$_8$ at 100 °C

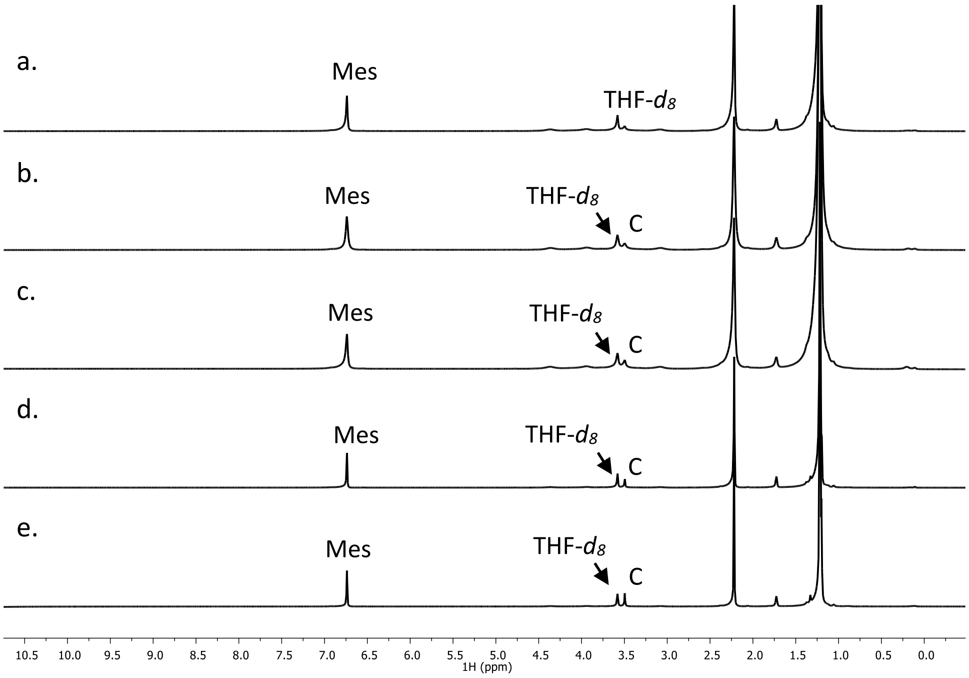


**Figure S.5.** $^1$H NMR reaction monitoring. Reaction conditions: **Mn1** (2.24 x $10^{-3}$ mmol), HBpin (0.224 mmol), $CO_2$ (1 bar), 100 °C, THF-$d_8$ (0.4 mL), mesitylene as internal standard (0.056 mmol). Reaction time: a) 1 h; b) 3 h; c) 5 h; d) 24 h; e) 48 h.

## 5.1.6. $CO_2$ hydroboration in dmso-$d_6$ at 25 °C

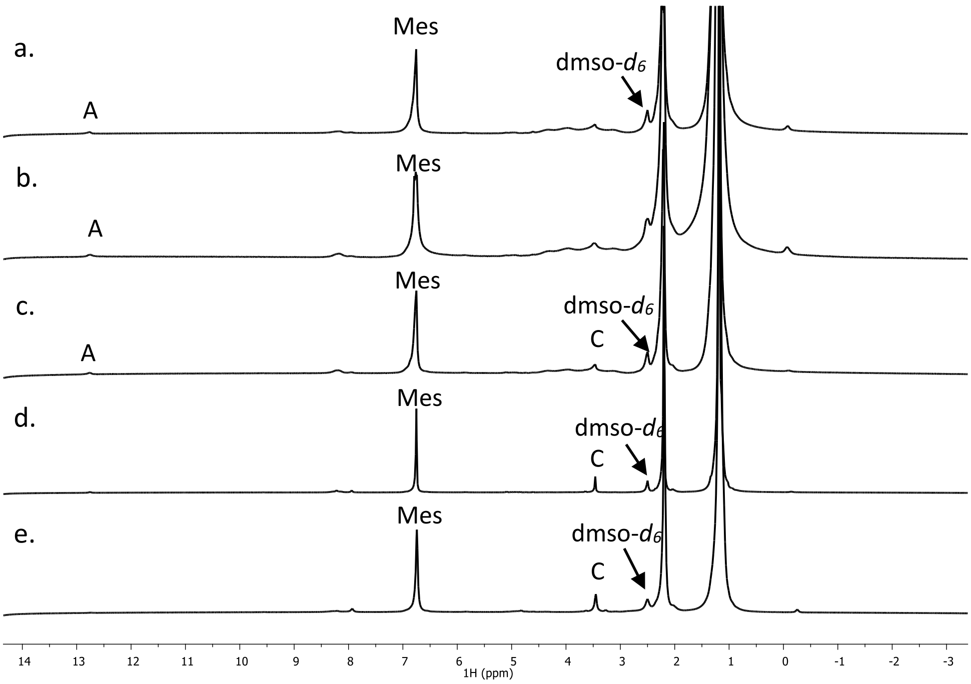


**Figure S.6.** $^1$H NMR reaction monitoring. Reaction conditions: **Mn1** (2.24 x $10^{-3}$ mmol), HBpin (0.224 mmol), $CO_2$ (1 bar), RT, dmso-$d_6$ (0.4 mL), mesitylene as internal standard (0.056 mmol). Reaction time: a) 1 h; b) 3 h; d) 5 h; d) 24 h; e) 48 h.

## 5.1.7. $CO_2$ hydroboration in dmso-*d₆* at 40 °C

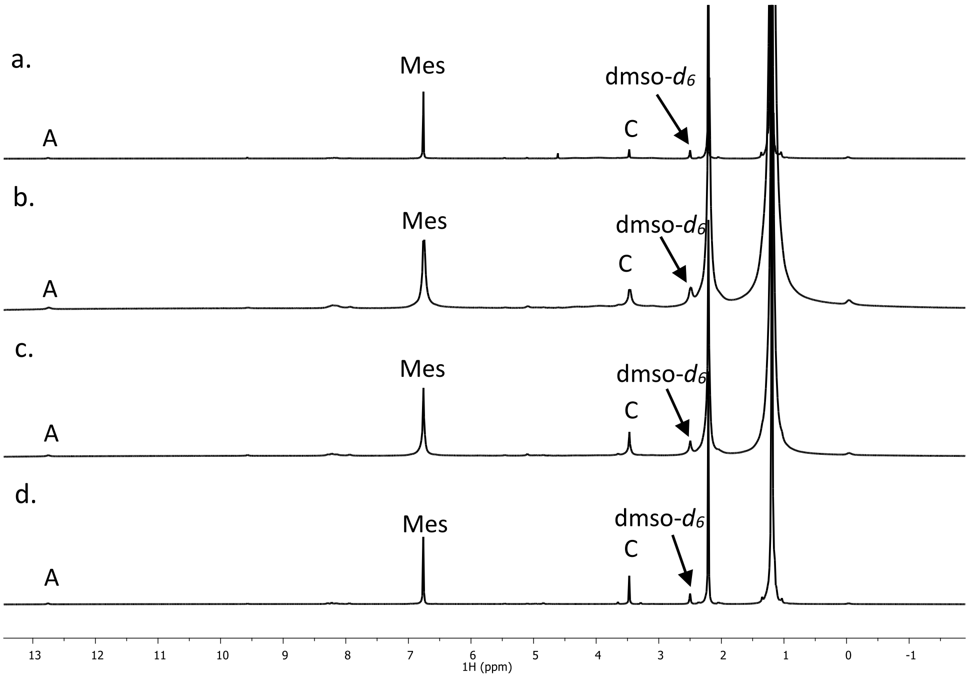


**Figure S.7.** $^1H$ NMR reaction monitoring. Reaction conditions: **Mn1** ($2.24 \times 10^{-3}$ mmol), HBpin (0.224 mmol), $CO_2$ (1 bar), 40 °C, dmso-$d_6$ (0.4 mL), mesitylene as internal standard (0.056 mmol). Reaction time: a) 1 h; b) 3 h; c) 5 h; d) 24 h.

## 5.1.8. $CO_2$ hydroboration in dmso-$d_6$ at 60 °C

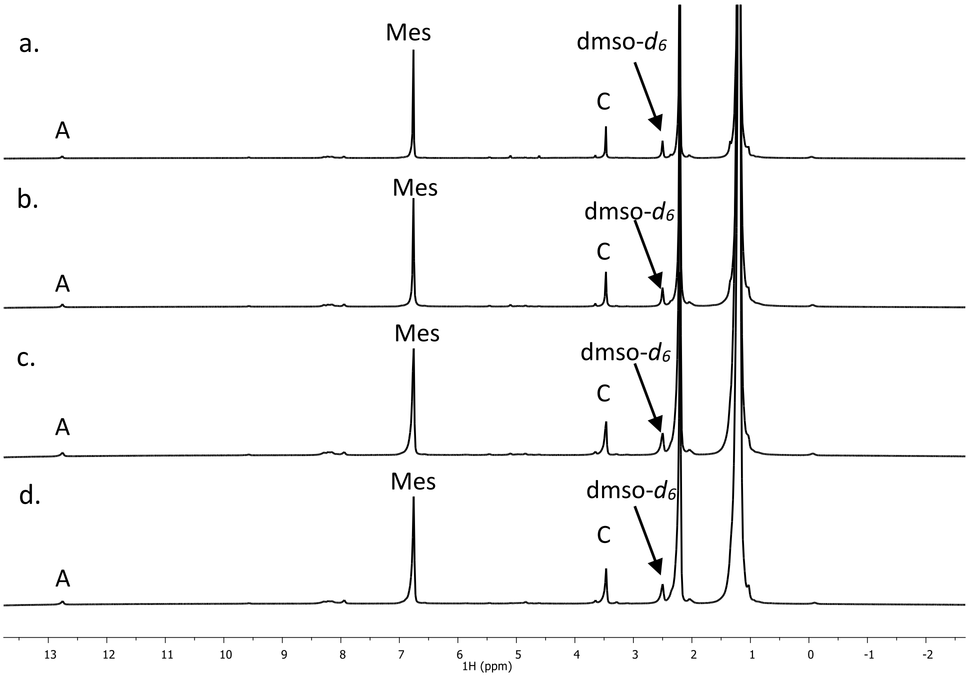


**Figure S.8.** $^1H$ NMR reaction monitoring. Reaction conditions: **Mn1** ($2.24 \times 10^{-3}$ mmol), HBpin (0.224 mmol), $CO_2$ (1 bar), 60 °C, dmso-$d_6$ (0.4 mL), mesitylene as internal standard (0.056 mmol). Reaction time: a) 1 h; b) 3 h; c) 5 h; d) 24 h.

## 5.2. $CO_2$ hydroboration in the presence of Mn2

### 5.2.1. $CO_2$ hydroboration in dmso-$d_6$ at 40 °C

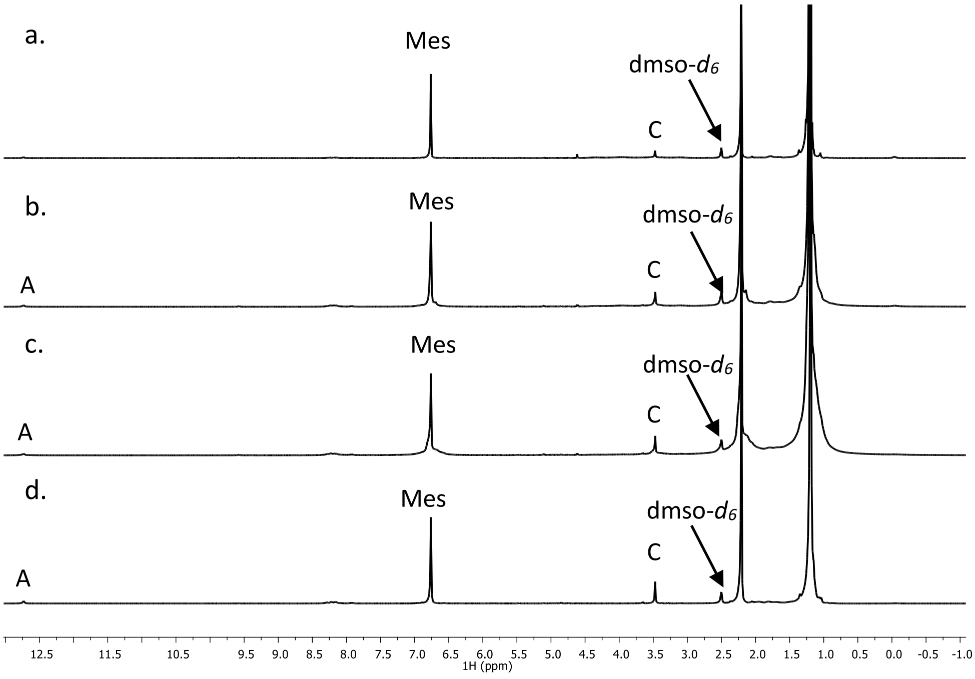


**Figure S.9.** $^1$H NMR reaction monitoring. Reaction conditions: **Mn2** ($2.24 \times 10^{-3}$ mmol), HBpin (0.224 mmol), $CO_2$ (1 bar), 40 °C, dmso-$d_6$ (0.4 mL), mesitylene as internal standard (0.056 mmol). Reaction time: a) 1 h; b) 3 h; c) 5 h; d) 24 h.

### 5.2.2. $CO_2$ hydroboration in dmso-$d_6$ at 60 °C

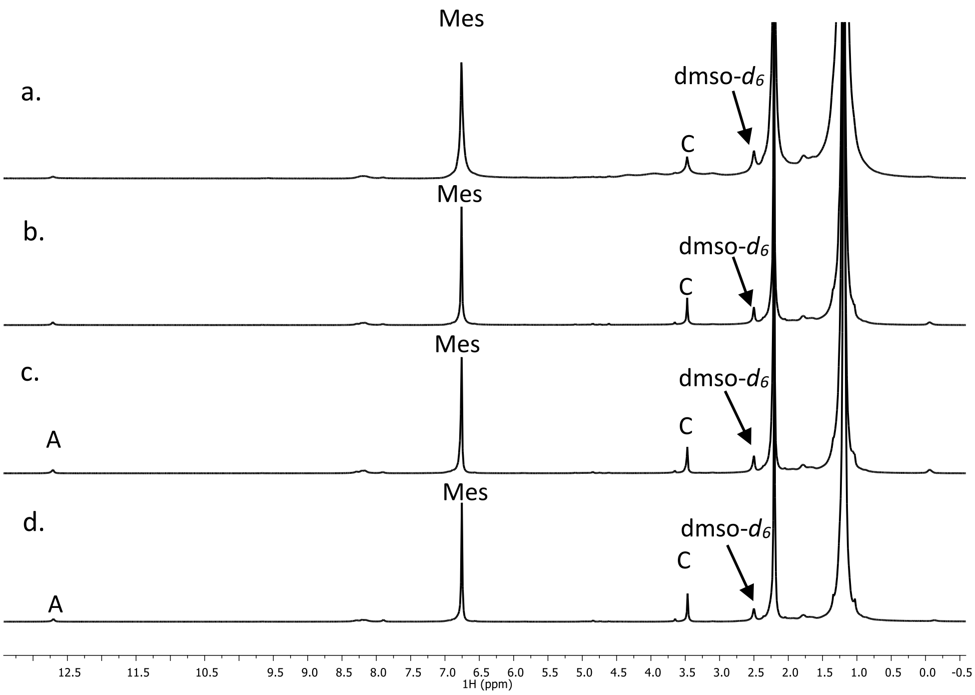


**Figure S.10.** $^1$H NMR reaction monitoring. Reaction conditions: **Mn2** ($2.24 \times 10^{-3}$ mmol), HBpin (0.224 mmol), $CO_2$ (1 bar), 60 °C, dmso-$d_6$ (0.4 mL), mesitylene as internal standard (0.056 mmol). Reaction time: a) 1 h; b) 3 h; c) 5 h; d) 24 h.

## 5.3. $CO_2$ hydroboration in the presence of Mn3

### 5.3.1. $CO_2$ hydroboration in dmso-$d_6$ at 40 °C

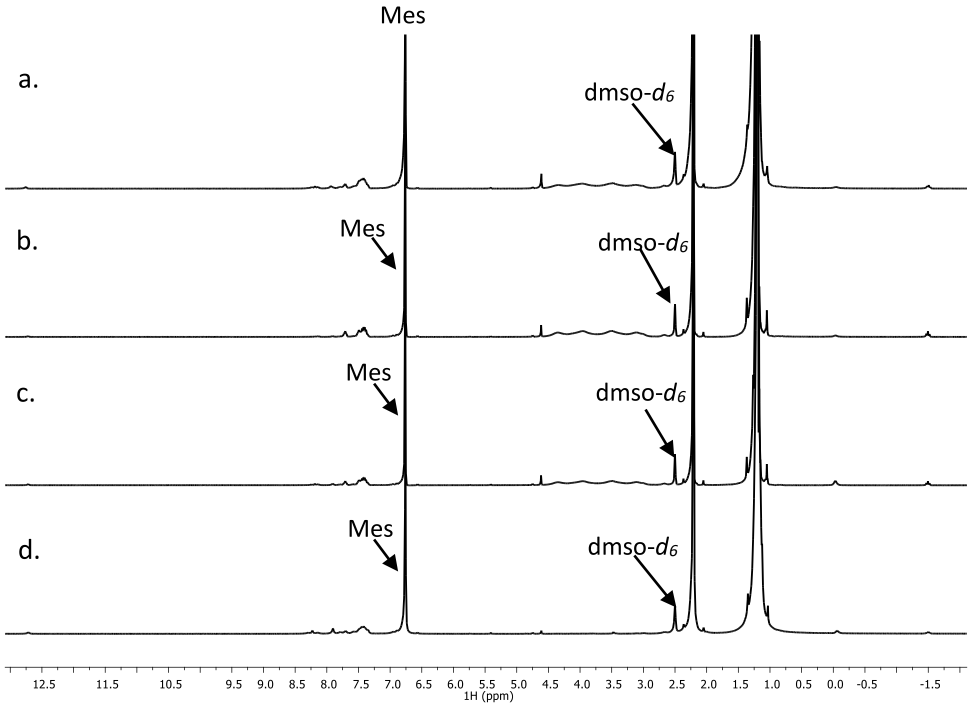


**Figure S.11.** $^1$H NMR reaction monitoring. Reaction conditions: **Mn3** ($2.24 \times 10^{-3}$ mmol), HBpin (0.224 mmol), $CO_2$ (1 bar), 40 °C, dmso-$d_6$ (0.4 mL), mesitylene as internal standard (0.056 mmol). Reaction time: a) 1 h; b) 3 h; c) 5 h; d) 24 h.

### 5.3.2. $CO_2$ hydroboration in dmso-$d_6$ at 60 °C

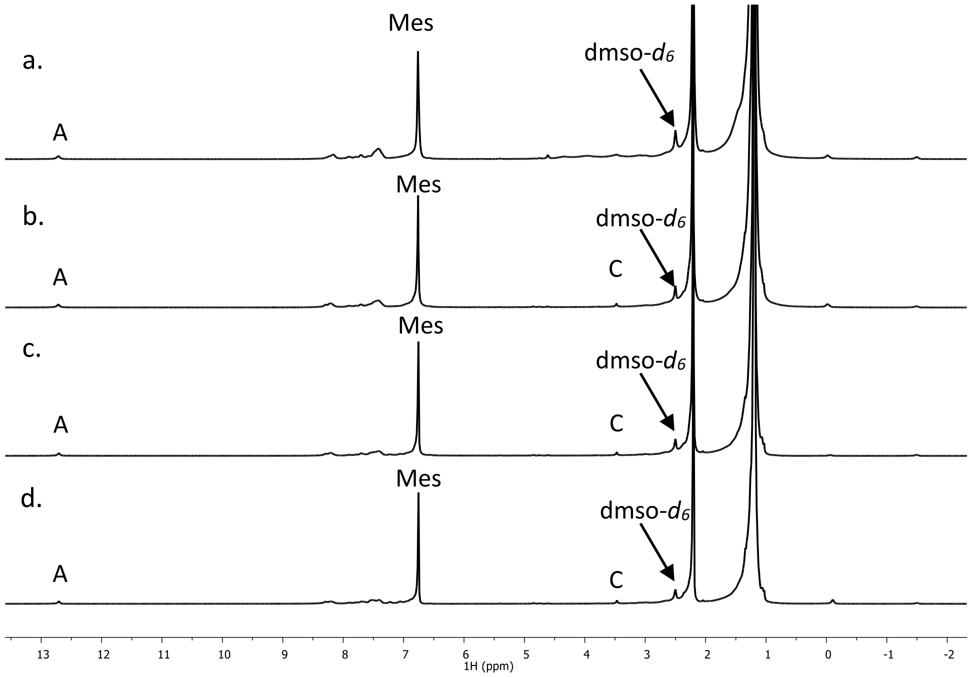


**Figure S.12.** $^1$H NMR reaction monitoring. Reaction conditions: **Mn3** ($2.24 \times 10^{-3}$ mmol), HBpin (0.224 mmol), $CO_2$ (1 bar), 60 °C, dmso-$d_6$ (0.4 mL), mesitylene as internal standard (0.056 mmol). Reaction time: a) 1 h; b) 3 h; c) 5 h; d) 24 h.

## 5.4. $CO_2$ hydroboration in the presence of Mn4

### 5.4.1. $CO_2$ hydroboration in dmso-$d_6$ at 40 °C

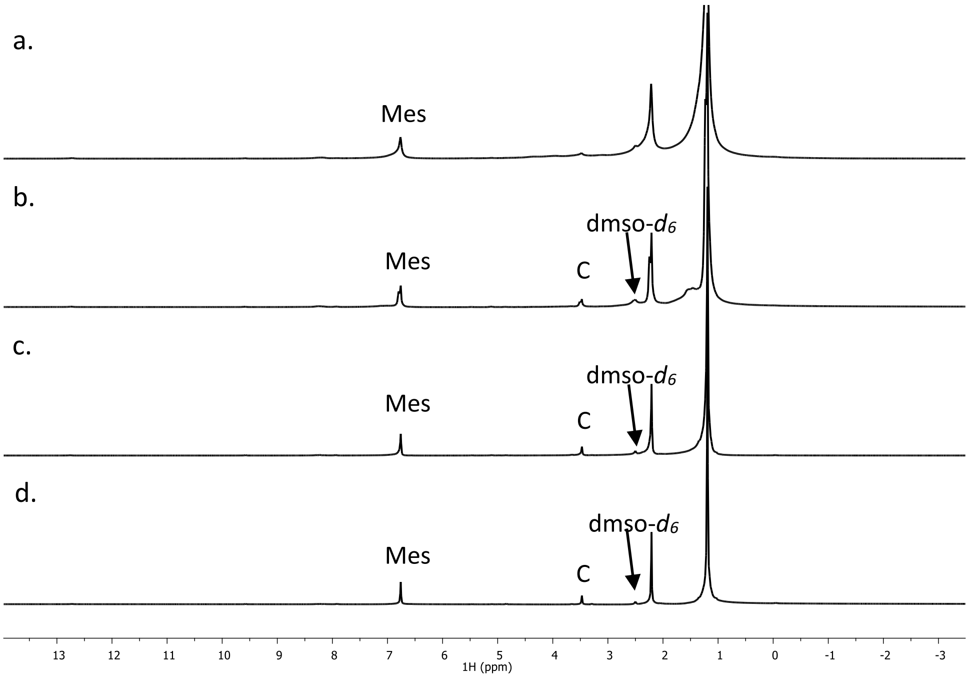


**Figure S.13.** $^1$H NMR reaction monitoring. Reaction conditions: **Mn4** (2.24 x $10^{-3}$ mmol), HBpin (0.224 mmol), $CO_2$ (1 bar), 40 °C, dmso-$d_6$ (0.4 mL), mesitylene as internal standard (0.056 mmol). Reaction time: a) 1 h; b) 3 h; c) 5 h; d) 24 h.

### 5.4.2. $CO_2$ hydroboration in dmso-$d_6$ at 60 °C

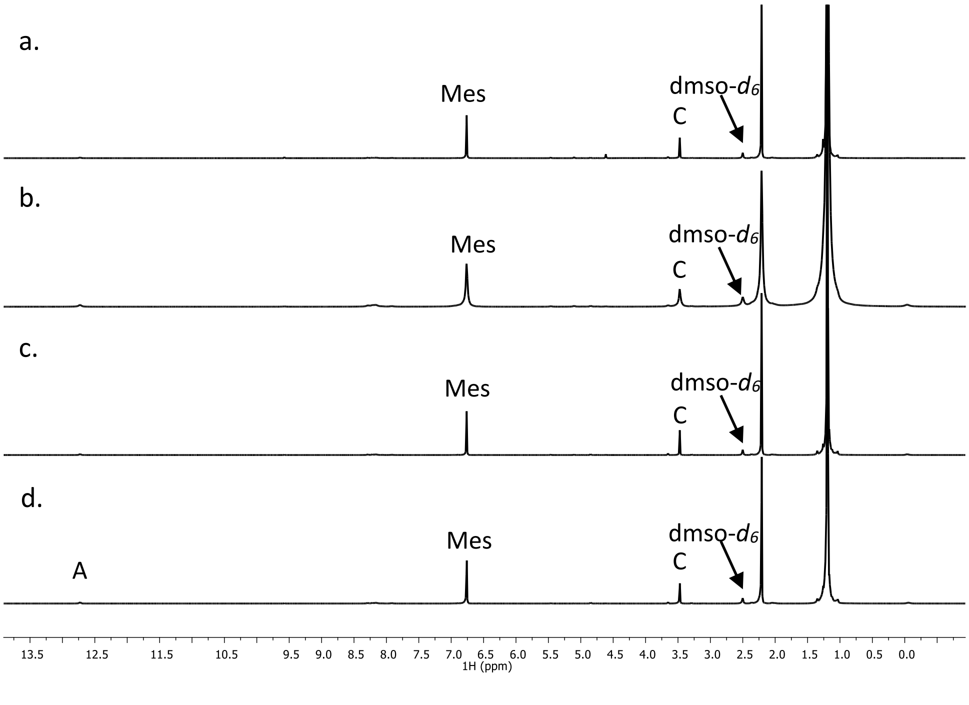


**Figure S.14.** $^1$H NMR reaction monitoring. Reaction conditions: **Mn4** (2.24 x $10^{-3}$ mmol), HBpin (0.224 mmol), $CO_2$ (1 bar), 60 °C, dmso-$d_6$ (0.4 mL), mesitylene as internal standard (0.056 mmol). Reaction time: a) 1 h; b) 3 h; c) 5 h; d) 24 h.

## 5.5. $CO_2$ hydroboration in the presence of Mn5

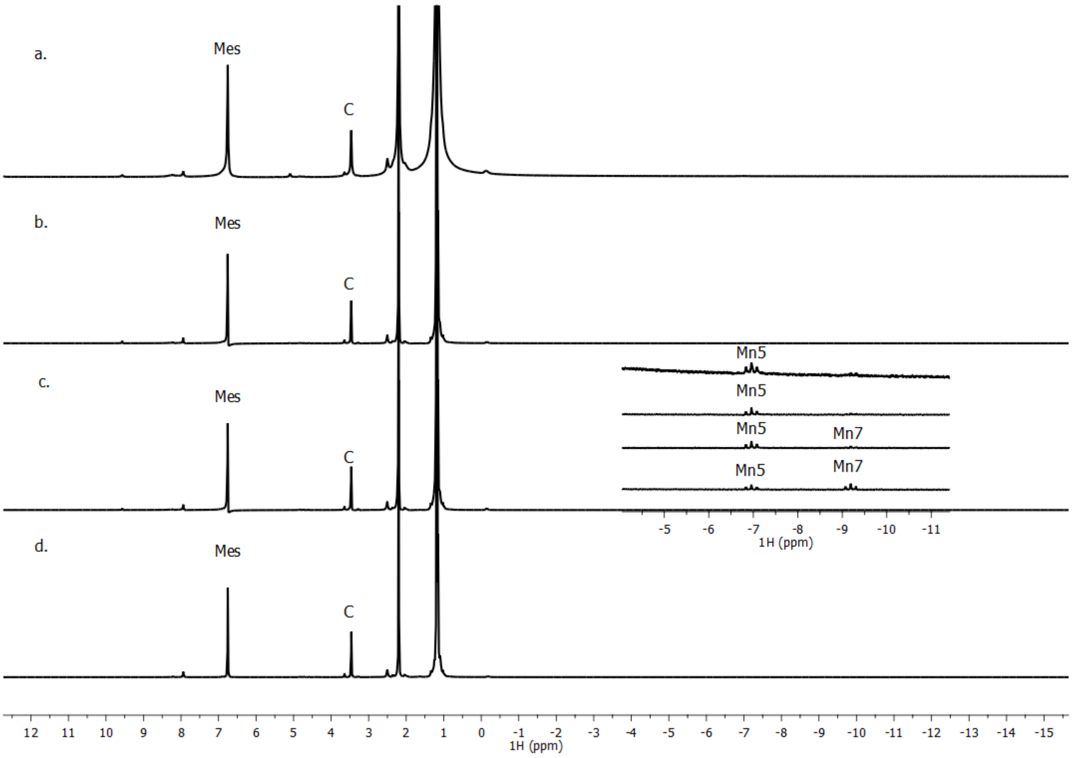


**Figure S.15.** $^{1}$H NMR reaction monitoring. Reaction conditions: **Mn5** ($2.24 \times 10^{-3}$ mmol), HBpin (0.224 mmol), $CO_2$ (1 bar), 60 °C, dmso-$d_6$ (0.4 mL), mesitylene as internal standard (0.056 mmol). Reaction time: a) 1 h; b) 3 h; c) 5 h; d) 24 h.

## 5.6. Blank test

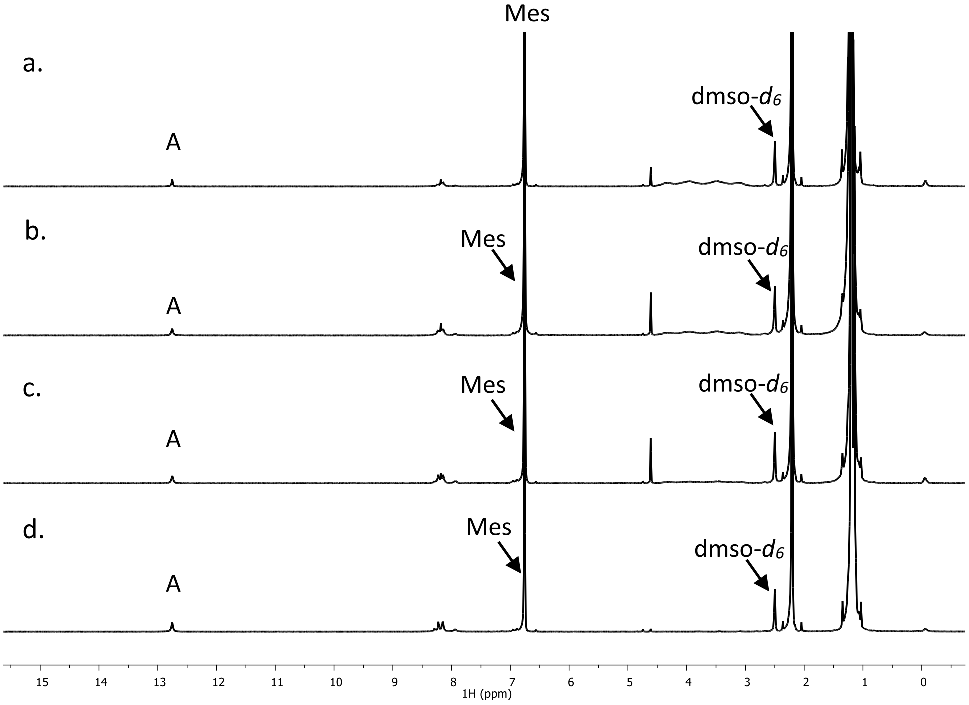


**Figure S.16.** $^{1}$H NMR reaction monitoring. Reaction conditions: HBpin (0.224 mmol), $CO_2$ (1 bar), 60 °C, dmso-$d_6$ (0.4 mL), mesitylene as internal standard (0.056 mmol). Reaction time: a) 1 h; b) 3 h; c) 5 h; d) 24 h.

## 5.7. $^{31}P\{^{1}H\}$ NMR monitoring of the catalytic run in the presence of Mn1

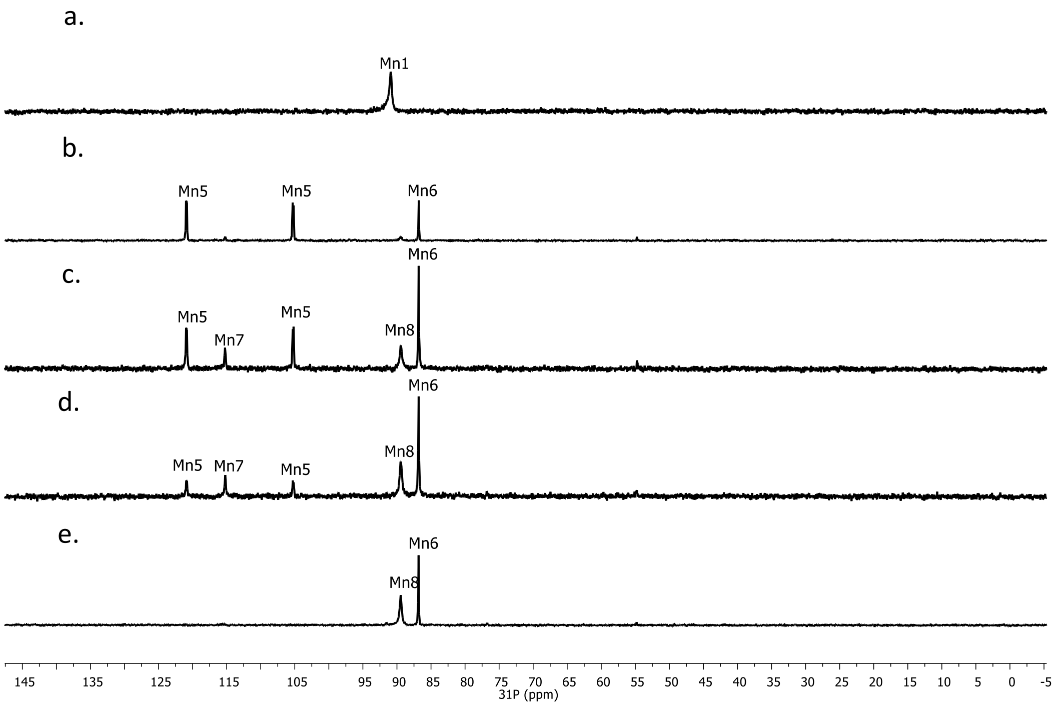


**Figure S.17.** $^{31}P\{^{1}H\}$ NMR reaction monitoring. Reaction conditions: **Mn1** ($2.24 \times 10^{-3}$ mmol), HBpin (0.224 mmol), $CO_2$ (1 bar), 40 °C, dmso-$d_6$ (0.4 mL), mesitylene as internal standard (0.056 mmol). a) **Mn1** before reaction; b) 1 h; c) 3 h; d) 5 h; e) 24 h.

## 5.8. $^1$H NMR after the catalytic run in the presence of Mn1

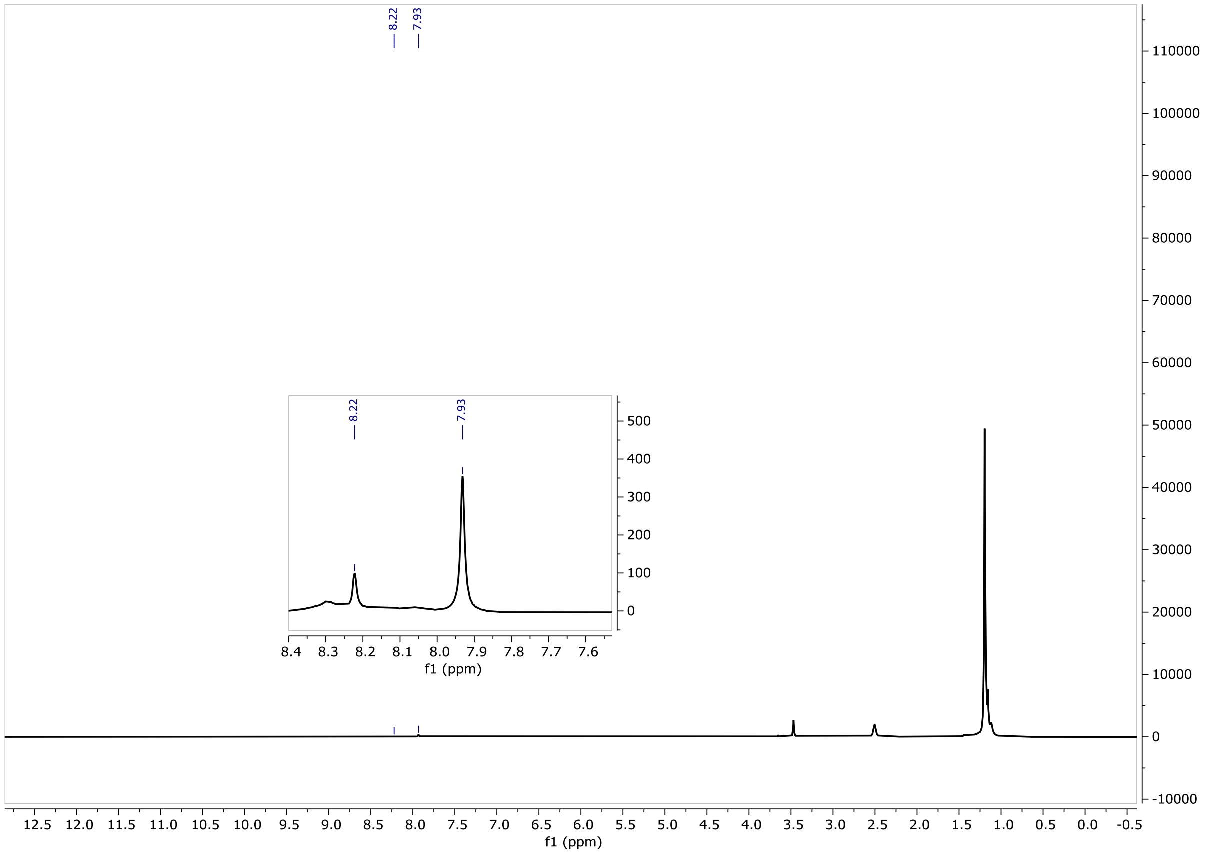


**Figure S.18.** $^1$H NMR after reaction. Reaction conditions: **Mn1** ($2.24 \times 10^{-3}$ mmol), HBpin (0.224 mmol), $CO_2$ (1 bar), 60 °C, dmso-$d_6$ (0.4 mL) after 16 h.

### 5.9. $^{31}P\{^1H\}$ NMR monitoring of the catalytic run in the presence of Mn4

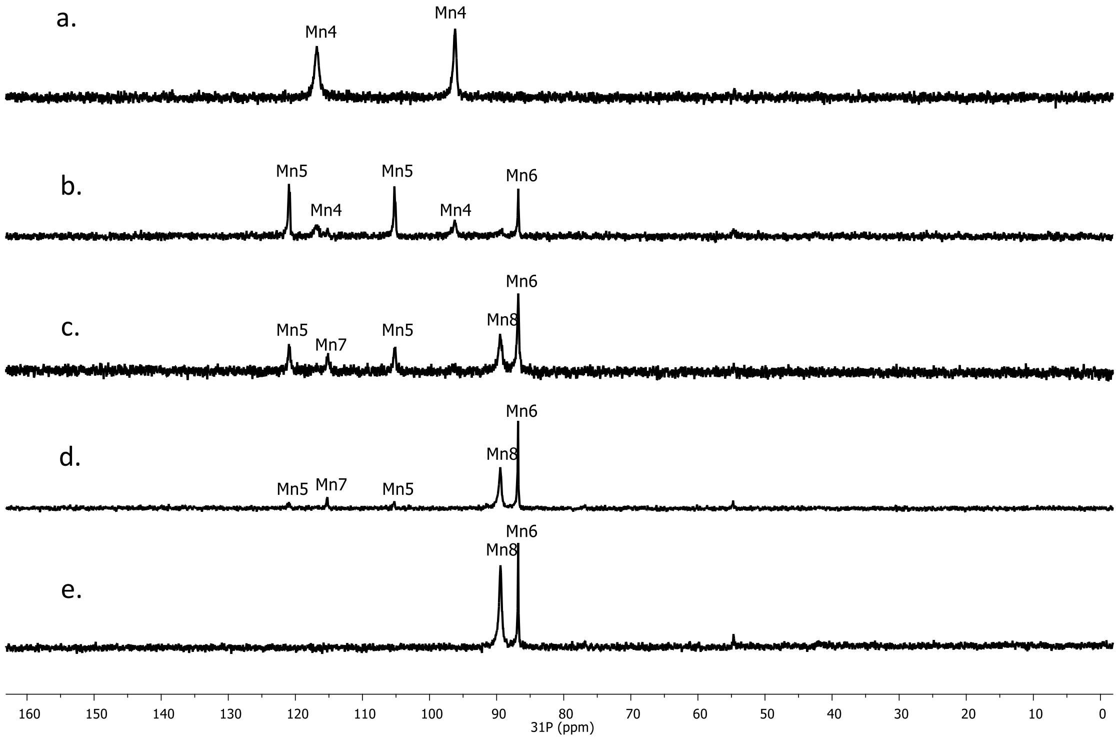


**Figure S.19.** $^{31}P\{^1H\}$ NMR reaction monitoring. Reaction conditions: **Mn4** (2.24 x $10^{-3}$ mmol), HBpin (0.224 mmol), $CO_2$ (1 bar), 40 °C, dmso-*d₆* (0.4 mL), mesitylene as internal standard (0.056 mmol). a) **Mn4** before reaction; b) 1 h; c) 3 h; d) 5 h; e) 24 h.

## 6. SELECTED NMR SPECTRA - MECHANISTIC STUDIES

### 6.1. Spectra of Mn5 and Mn8

a) NMR tube scale experiment for the *in situ* formation of **Mn5**

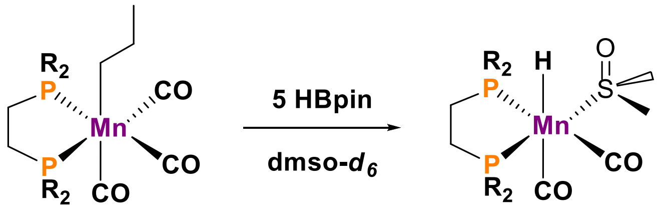


**Mn1** **Mn5**

A NMR tube was charged under an Ar atmosphere with dmso-$d_6$ (400 µL), pinacolborane (0.11 mmol) and **Mn1** in 5:1 ratio. The NMR tube was kept in an oil bath set to 60 °C for 1 h. After this time, the reaction mixture was analyzed by $^1H$ NMR and $^{31}P\{^1H\}$ NMR spectroscopy.

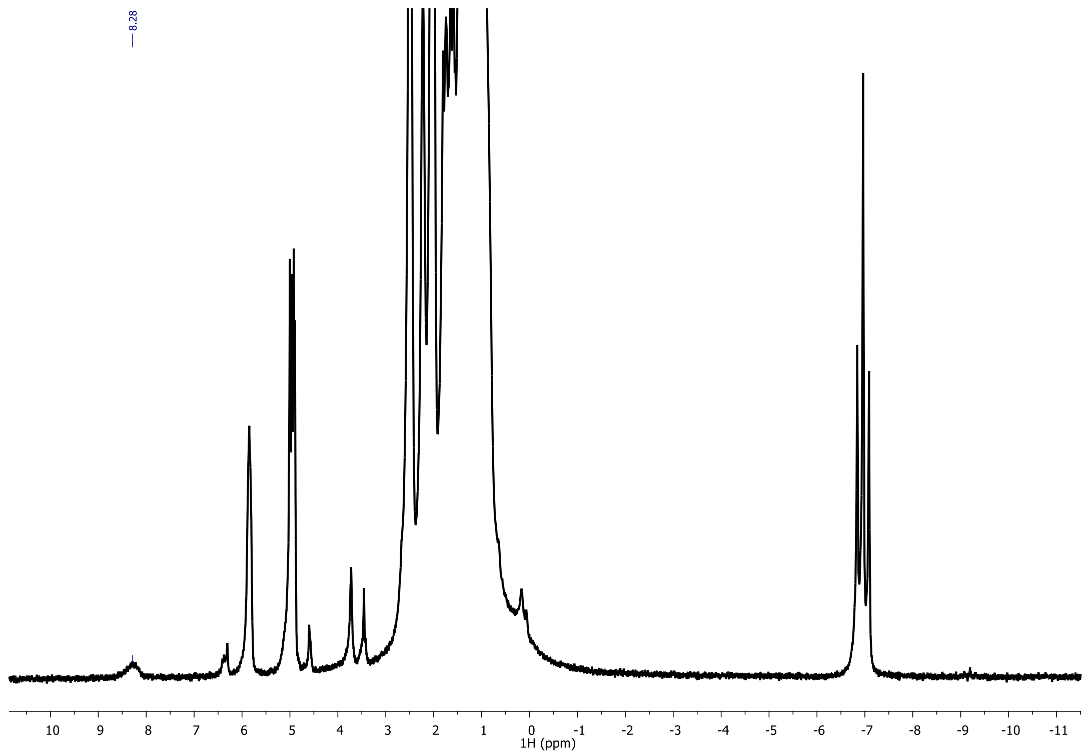


**Figure S.20.** $^{1}$H NMR spectrum of the reaction mixture giving **Mn5**. Reaction conditions: **Mn1** (2.24 x $10^{-3}$ mmol), HBpin (5 equiv.), 60 °C, dmso-$d_6$ (0.4 mL), 1 h, Ar atmosphere.

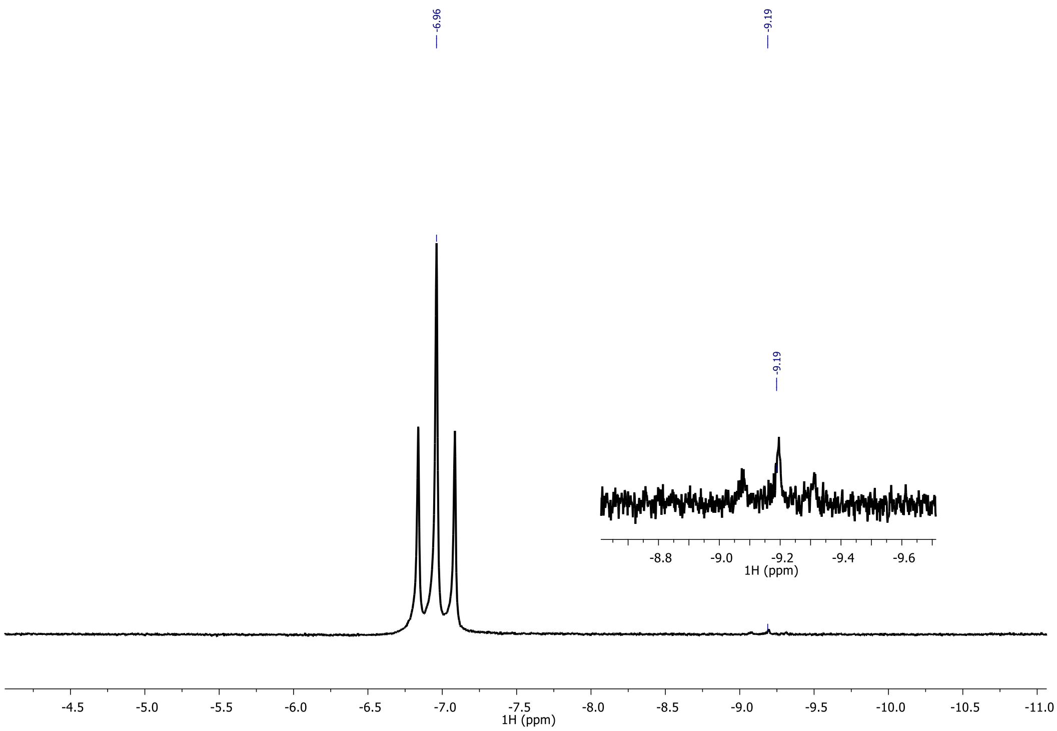


**Figure S.21.** $^{1}$H NMR spectrum of the reaction mixture giving **Mn5** negative chemical shift range (detail). Reaction conditions: **Mn1** (2.24 x $10^{-3}$ mmol), HBpin (5 equiv.), 60 °C, dmso-$d_6$ (0.4 mL), 1 h, Ar atmosphere.

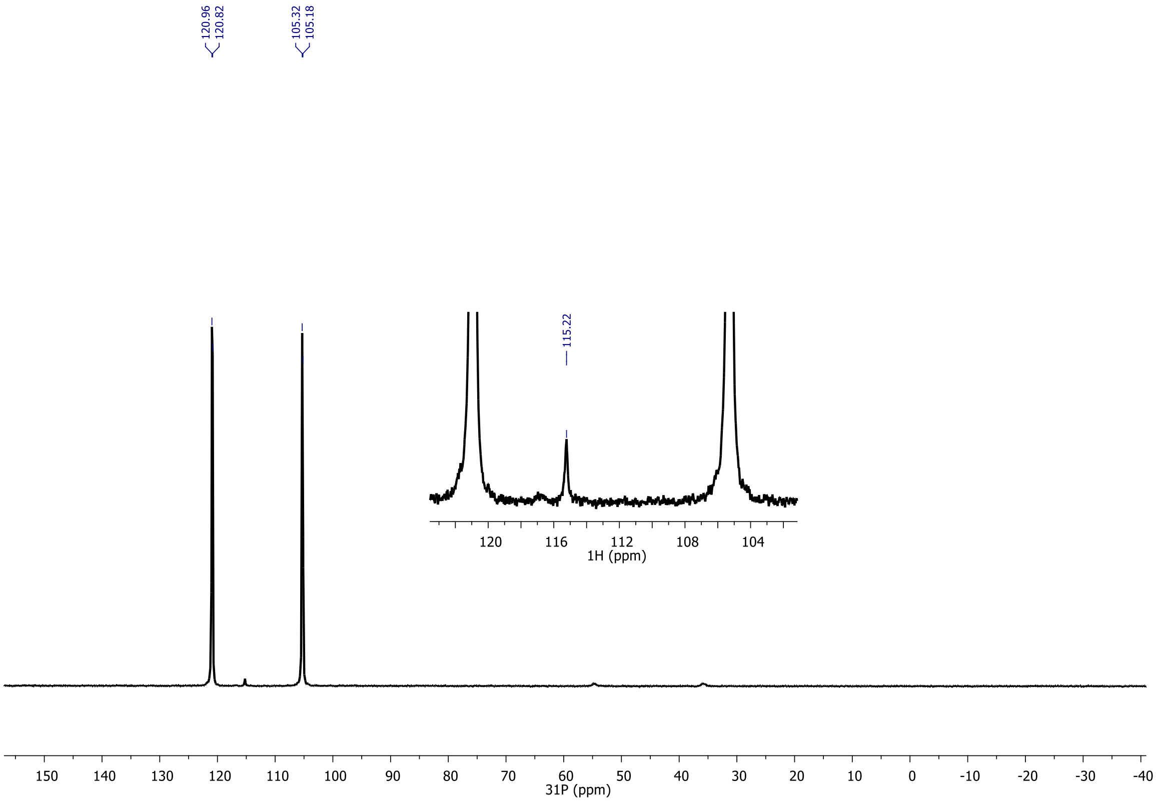


**Figure S.22.** $^{31}P\{^{1}H\}$ NMR spectrum of the reaction mixture giving **Mn5** with detail of the minor signal at 115.2 ppm. Reaction conditions: **Mn1** ($2.24 \times 10^{-3}$ mmol), HBpin (5 equiv.), 60 °C, dmso-$d_6$ (0.4 mL), 1 h, Ar atmosphere.

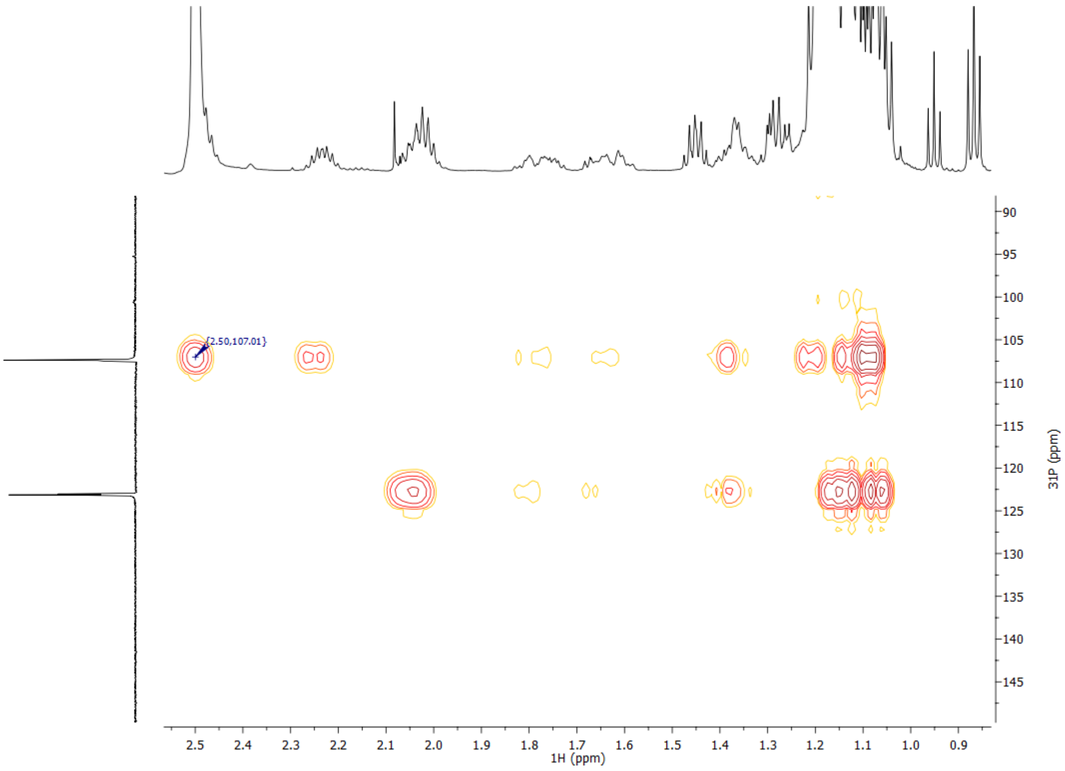


**Figure S.23.** [$^{1}H$, $^{31}P$]-HMQC NMR spectrum of the reaction mixture giving **Mn5**. The spectrum shows the coordination of dmso-$d_6$ to the Mn center. Reaction conditions: **Mn1** ($2.24 \times 10^{-3}$ mmol), HBpin (5 equiv.), 60 °C, dmso-$d_6$ (0.4 mL), mesitylene as internal standard (0.056 mmol), 1 h, Ar atmosphere.

b) NMR spectra of **Mn8**

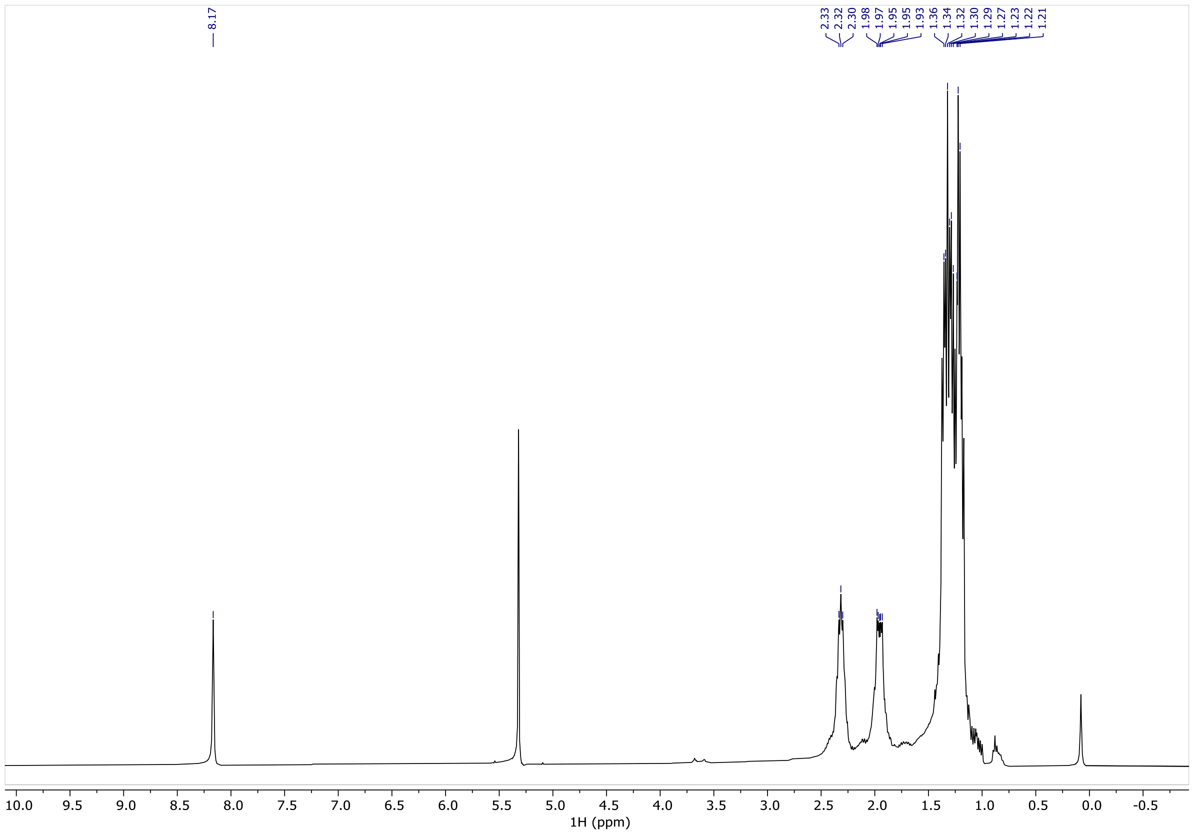


**Figure S.24.** $^{1}H$ NMR spectrum of isolated **Mn8** (see section 2.1.5).

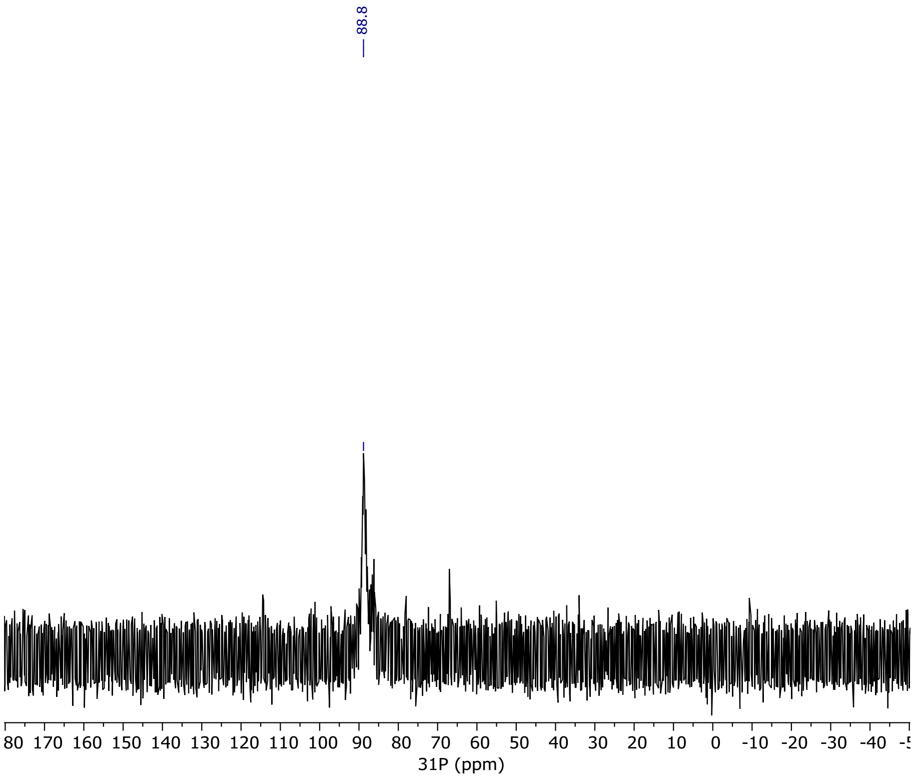


**Figure S.25.** $^{31}P\{^{1}H\}$ NMR spectrum of isolated **Mn8** (see section 2.1.5).

## 6.2. Reaction between Mn1 and HBpin (1:5) under $CO_2$ (1 bar)

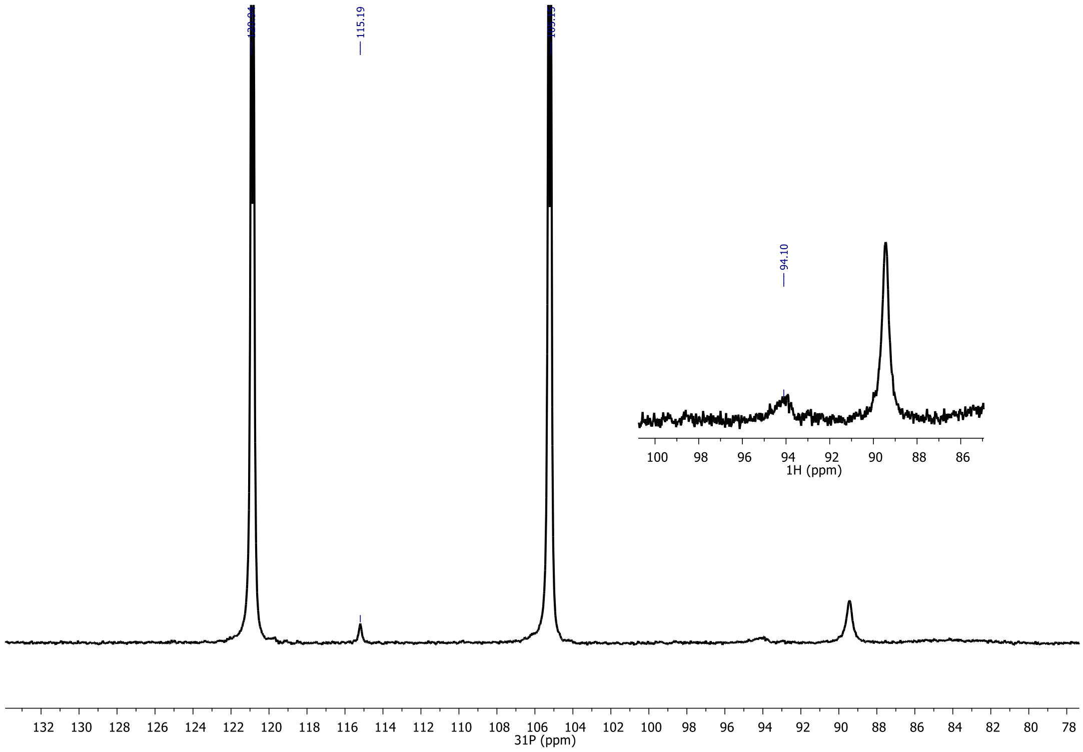


**Figure S.26.** $^{31}P\{^1H\}$ NMR spectrum after 24 h. Reaction conditions: **Mn1** ($2.24 \times 10^{-3}$ mmol), HBpin (5 equiv.), 60 °C, dmso-$d_6$ (0.4 mL), mesitylene as internal standard (0.056 mmol), $CO_2$ (1 bar), 24 h.

## 6.3. Reaction between Mn8 and HBpin (1:5) under $CO_2$ (1 bar)

**Mn8** ($2.24 \times 10^{-3}$ mmol) was dissolved in dmso-$d_6$ (0.4 mL) and HBPin (0.22 mmol) was added. In a NMR tube the solution was degassed via three freeze-pump-thaw cycles and the atmosphere was exchanged for $CO_2$ (1 bar). The tube was placed in an oil bath and kept at 60 °C overnight. The sample was analyzed by $^1H$, $^{31}P\{^1H\}$ and $^{11}B$ NMR spectroscopy.

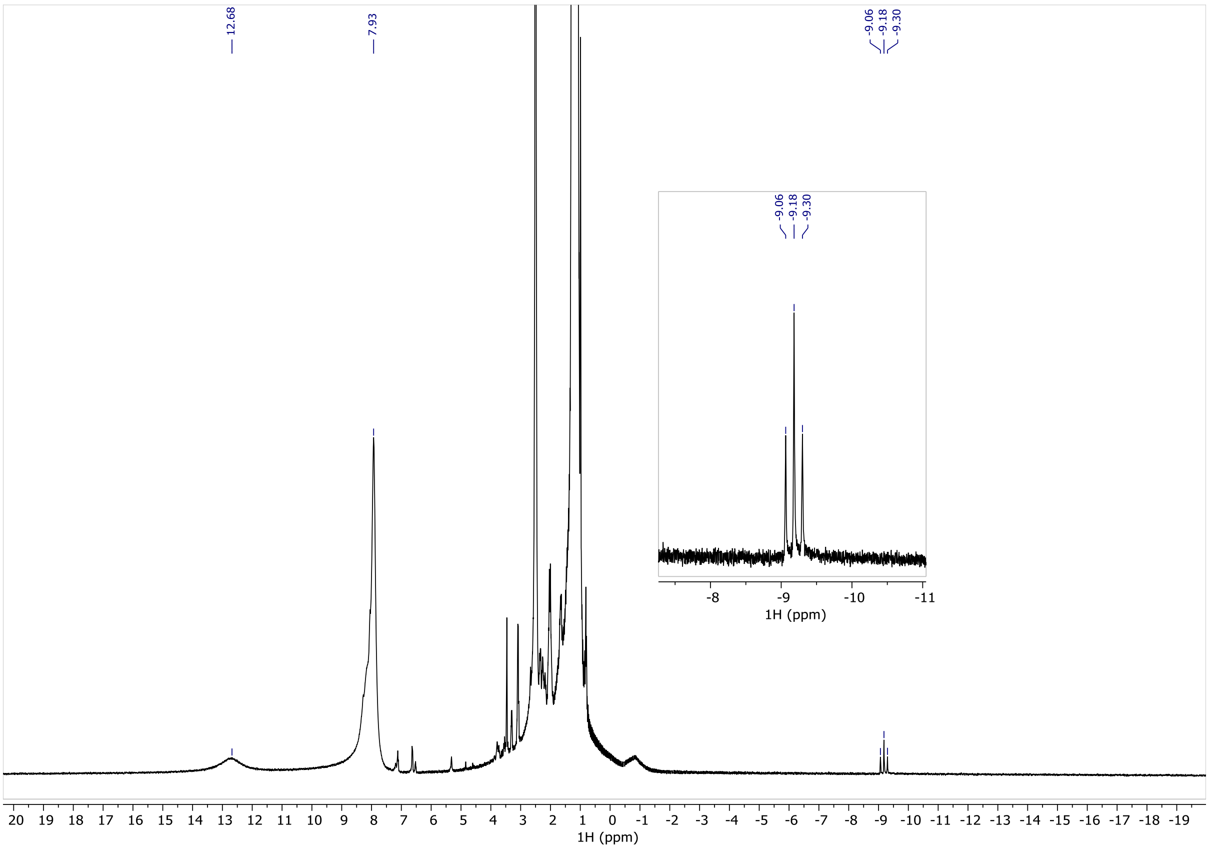


**Figure S.27.** $^1$H NMR spectrum after 24 h with detail of the negative chemical shift range. Reaction conditions: **Mn8** (2.24 x $10^{-3}$ mmol), HBpin (0.22 mmol mmol), 60 °C, dmso-$d_6$ (0.4 mL), $CO_2$ atmosphere.

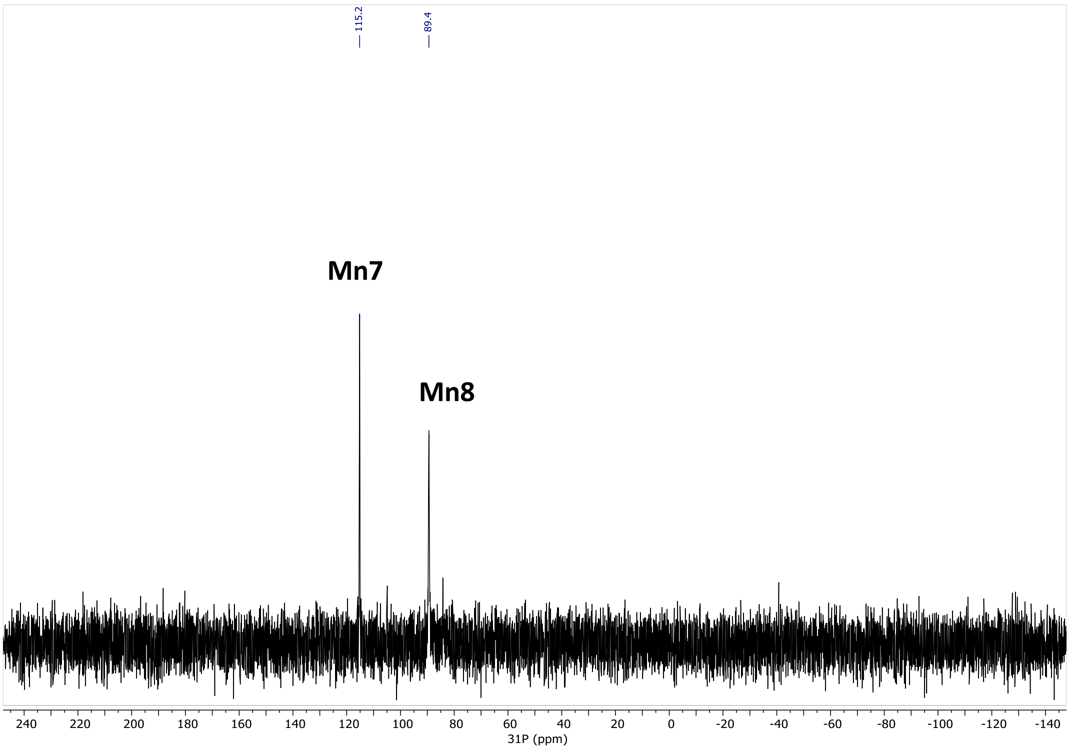


**Figure S.28.** $^{31}$P{$^1$H} NMR spectrum after 24 h. Reaction conditions: **Mn8** (2.24 x $10^{-3}$ mmol), HBpin (0.22 mmol mmol), 60 °C, dmso-$d_6$ (0.4 mL), $CO_2$ atmosphere.

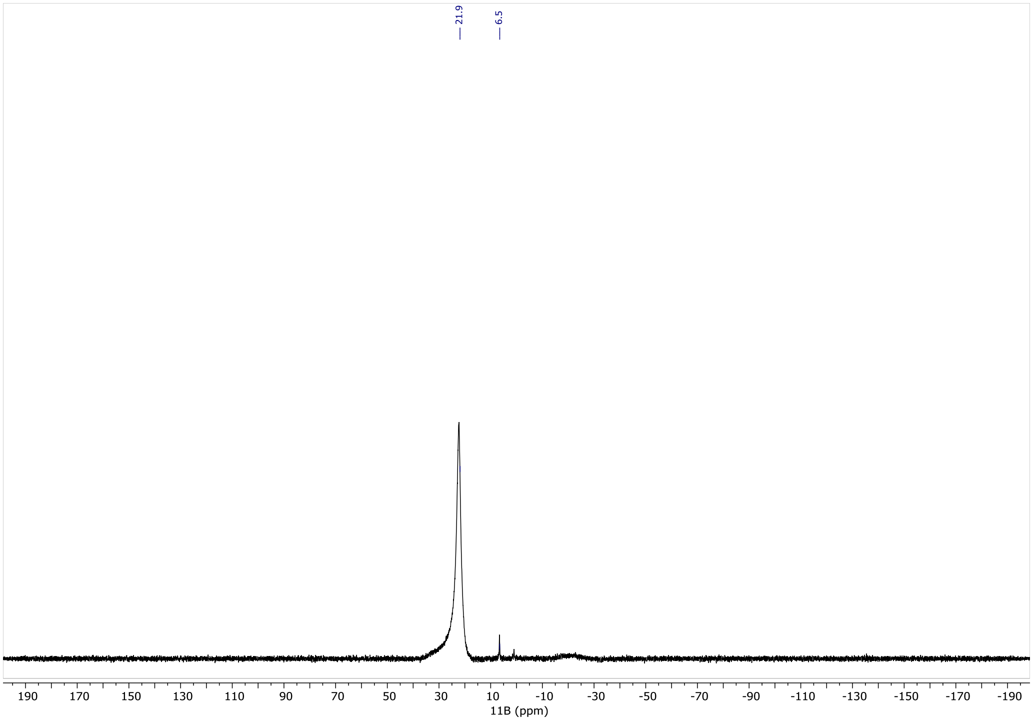


**Figure S.29.** $^{11}B$ NMR spectrum after 24 h. Reaction conditions: **Mn8** ($2.24 \times 10^{-3}$ mmol), HBpin (0.22 mmol mmol), 60 °C, dmso-$d_6$ (0.4 mL), $CO_2$ atmosphere.

## 6.4. Reaction between HCOOH and HBpin (1:1), in the absence of $CO_2$

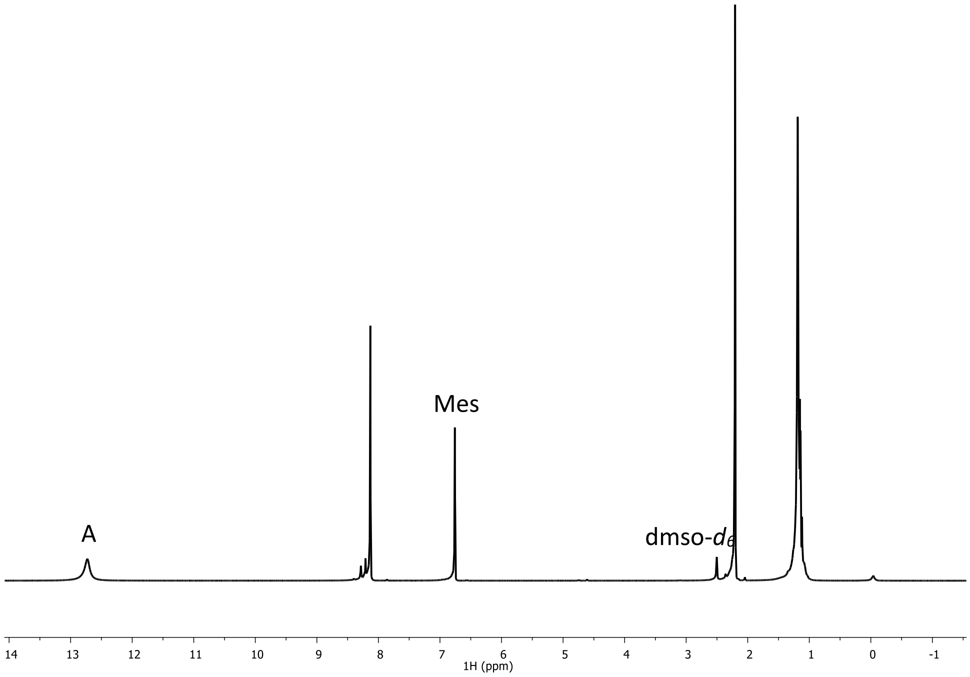


**Figure S.30.** $^{1}H$ NMR spectrum after 24 h. Reaction conditions: HBpin (0.224 mmol), HCOOH (0.224 mmol), 60 °C, dmso-$d_6$ (0.4 mL), mesitylene as internal standard (0.056 mmol), $N_2$ atmosphere, 24 h.

## 6.5. Reaction between HCOOH and HBpin (1:1), in the absence of $CO_2$

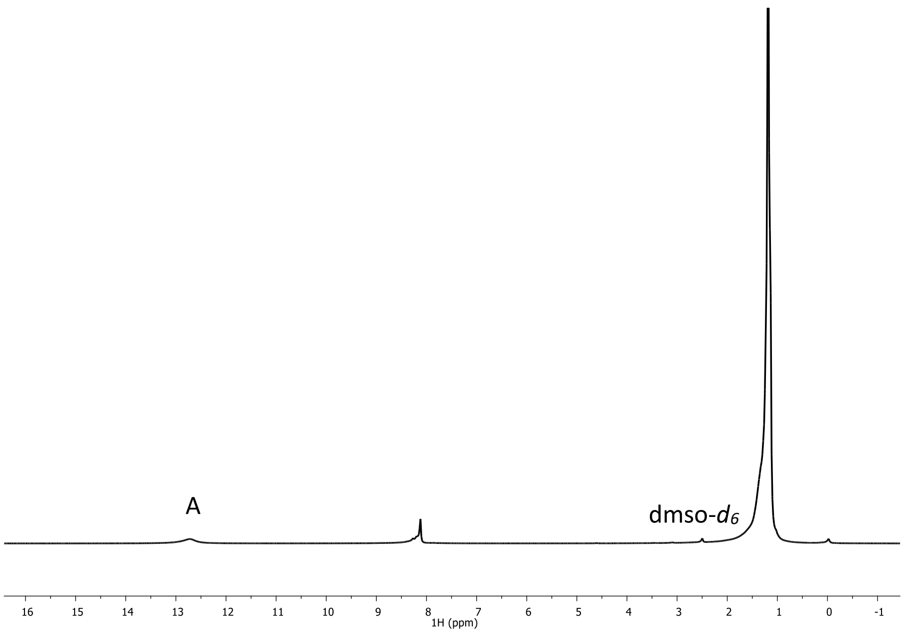


**Figure S.31.** Reaction conditions: HBpin (0.265 mmol), HCOOH (0.265mmol), 60 °C, dmso-$d_6$ (0.4 mL), mesitylene as internal standard (0.056 mmol), $N_2$ atmosphere, 24 h.

## 6.6. Reaction between HCOOH and HBpin (1:1) in the presence of Mn1.

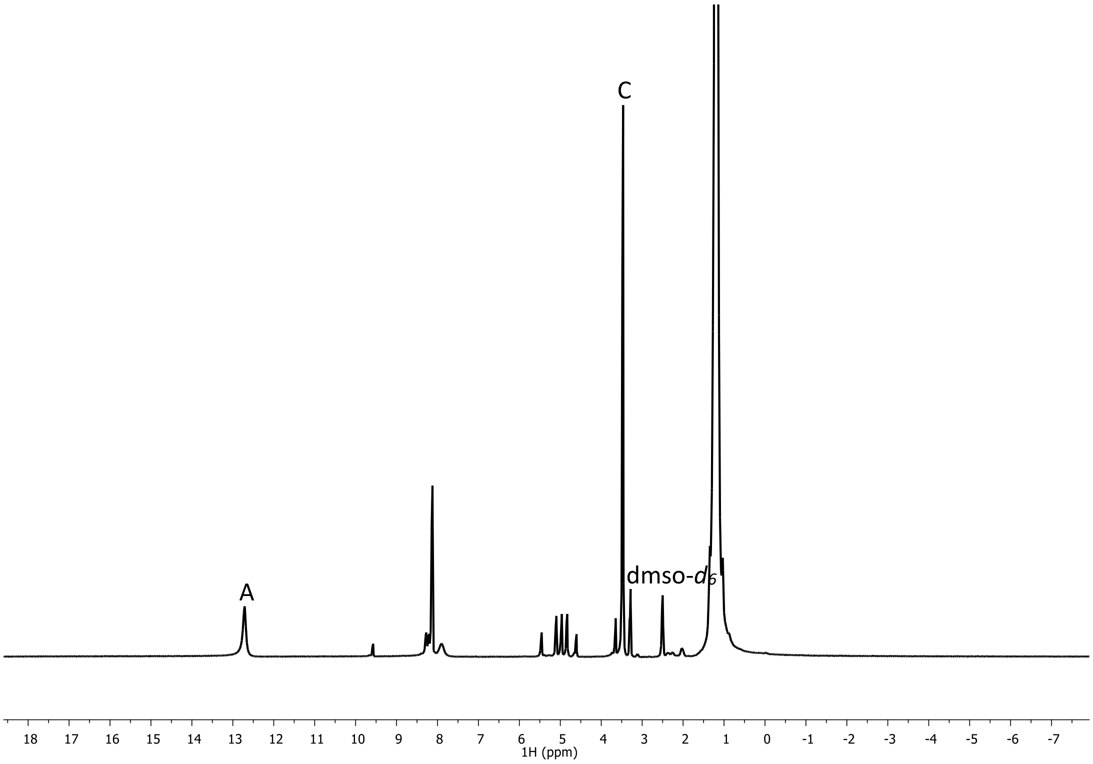


**Figure S.32.** $^1$H NMR spectrum after 24 h. Reaction conditions: **Mn1** (2.65 x $10^{-3}$ mmol), HBpin (0.265 mmol), HCOOH (0.265 mmol), 60 °C, dmso-$d_6$ (0.4 mL), mesitylene as internal standard (0.056 mmol), $N_2$ atmosphere, 24 h.

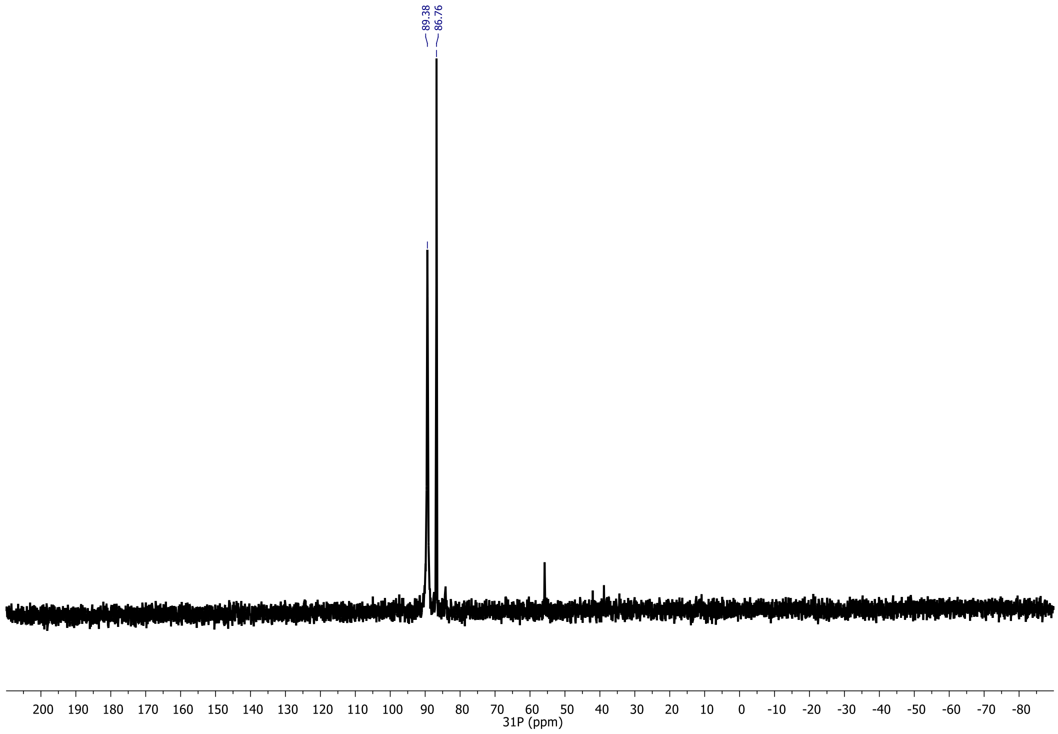


**Figure S.33.** $^{31}P\{^{1}H\}$ NMR spectrum after 24 h. Reaction conditions: **Mn1** (2.65 x $10^{-3}$ mmol), HBpin (0.265 mmol), HCOOH (0.265 mmol), 60 °C, dmso-*d*$_6$ (0.4 mL), mesitylene as internal standard (0.056 mmol), $N_2$ atmosphere, 24 h.

## 6.7. Evidence for the presence of (O-borylated) butanol

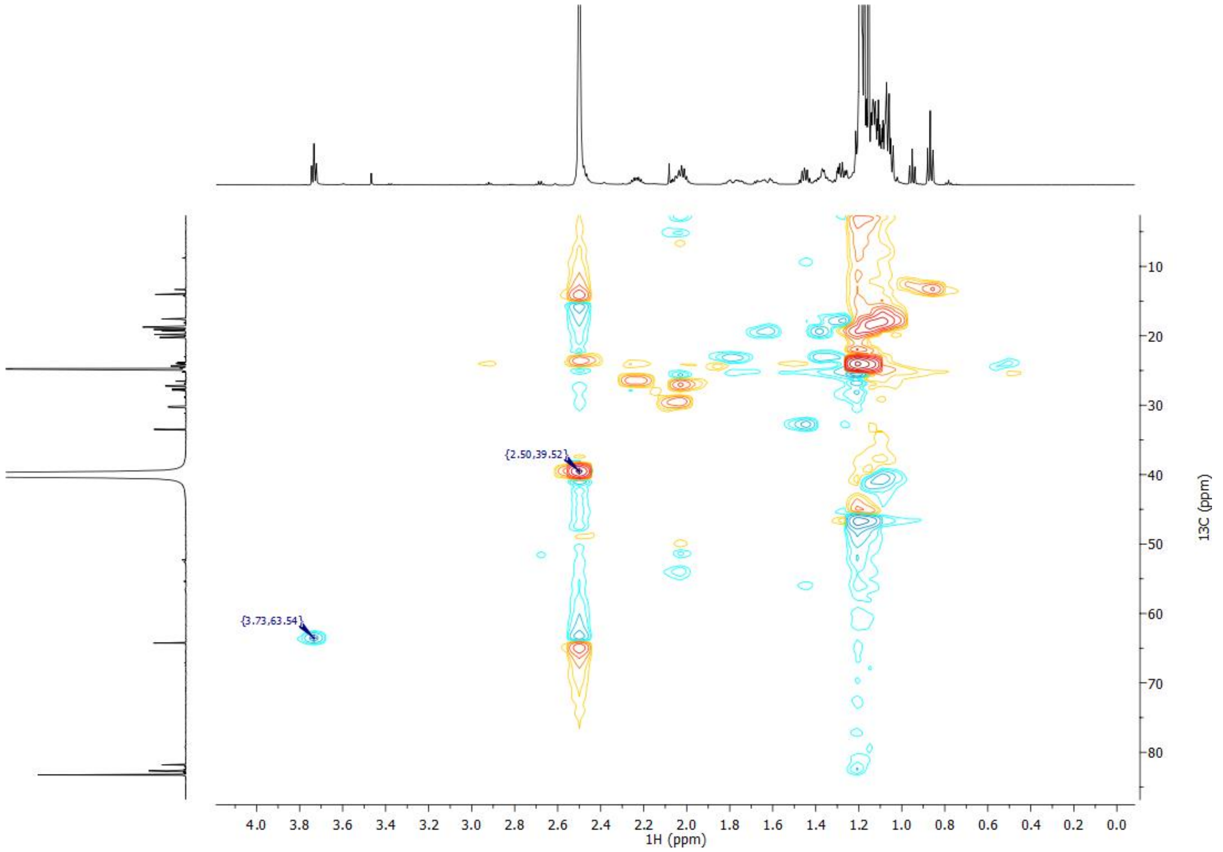


**Figure S.34.** [$^1$H, $^{13}$C]-HSQC NMR spectrum showing the presence of butanol under the reaction conditions (at 3.73 ppm ($^1$H), 63.54 ppm ($^{13}$C)). Reaction conditions: **Mn1** ($2.24 \times 10^{-3}$ mmol), HBpin (5 equiv.), 60 °C, dmso-$d_6$ (0.4 mL), mesitylene as internal standard (0.056 mmol), $N_2$ atmosphere.

## 6.8. NMR Spectra of Mn2

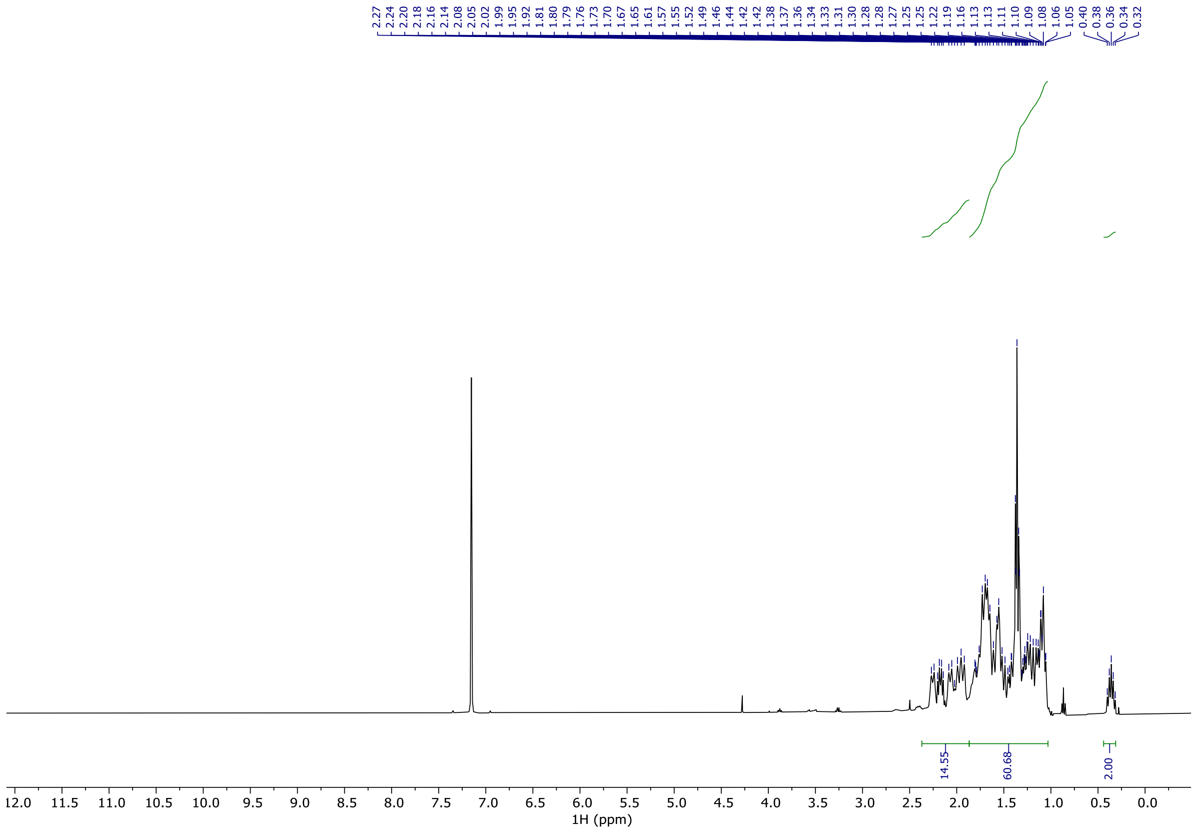


**Figure S.35.** $^{1}$H NMR spectrum of **Mn2**.

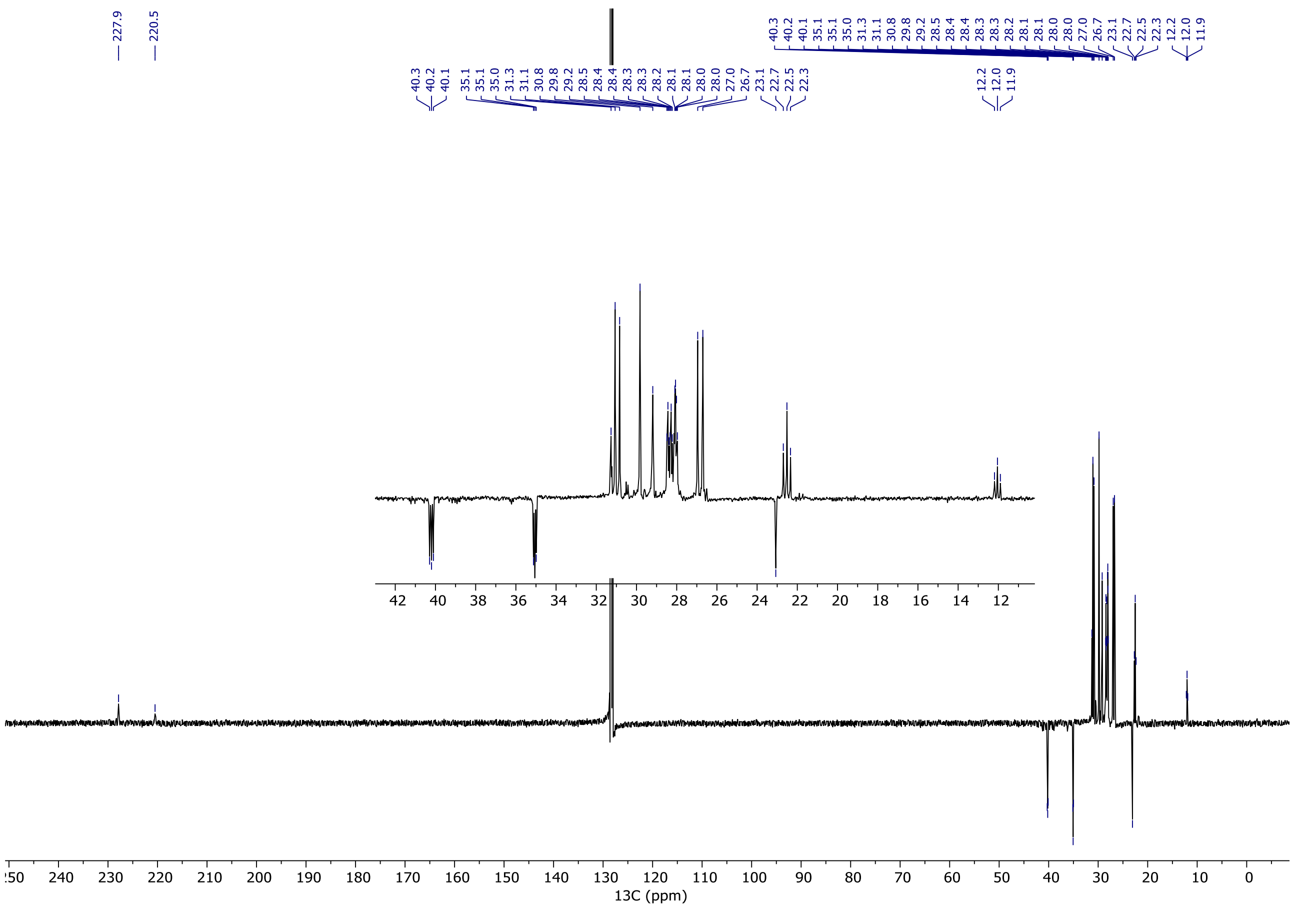


**Figure S.36.** $^{13}C\{^1H\}$ NMR spectrum of **Mn2**.

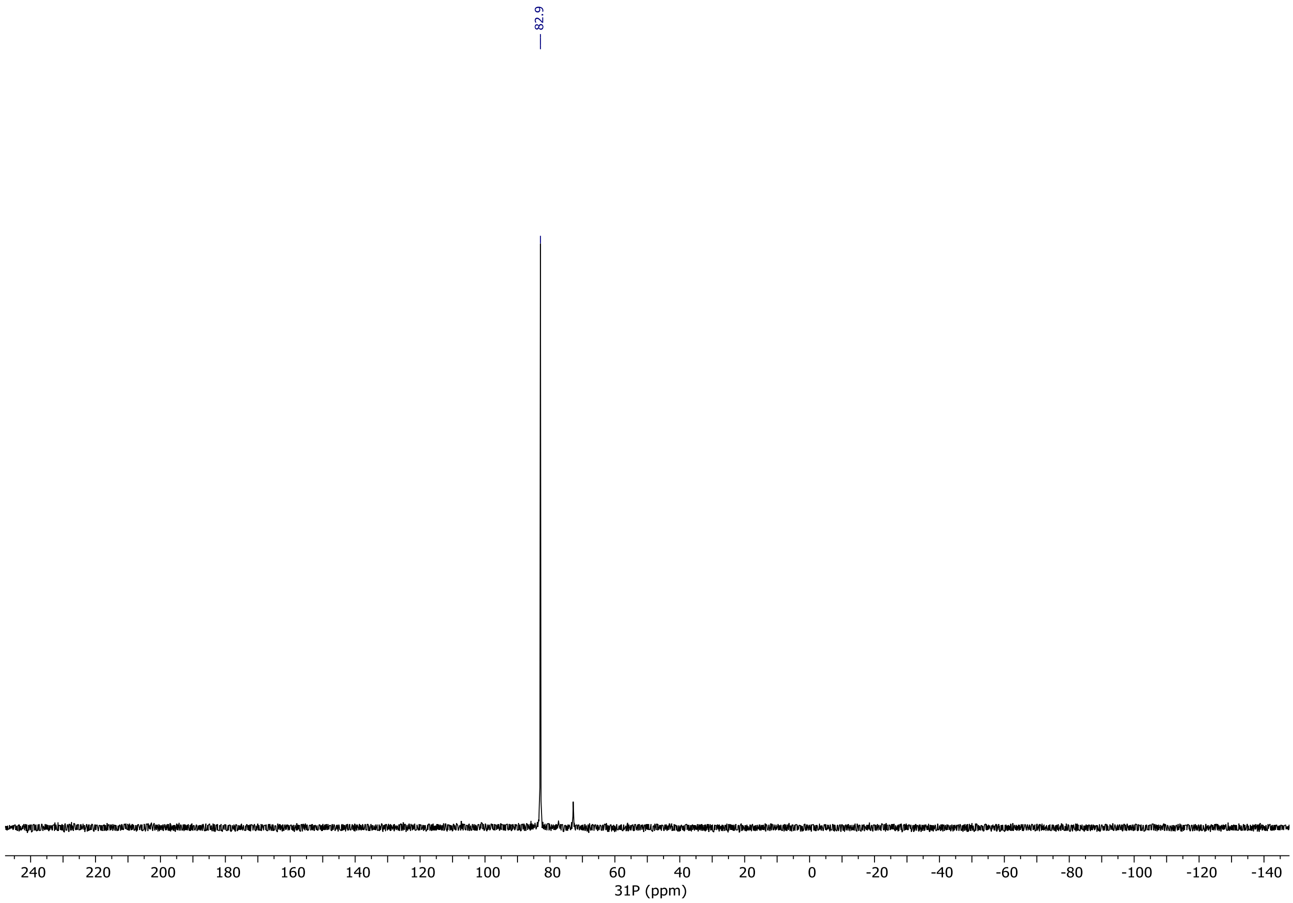


**Figure S.37.** $^{31}P\{^1H\}$ NMR spectrum of **Mn2**.

## 7. REFERENCES

(1) Weber, S.; Stöger, B.; Veiros, L. F.; Kirchner, K. Rethinking Basic Concepts - Hydrogenation of Alkenes Catalyzed by Bench-Stable Alkyl Mn(I) Complexes. *ACS Catal.* **2019**, *9*, 9715–9720.

(2) Kraihanzel, C. M.; Maples, P. K. Structures of Acyl- and Methylmanganese Carbonyl Complexes of Chelating Diphosphines. *J. Organometal. Chem.* **1969**, *20*, 269-272.

(3) Weber, S.; Zobernig, D.; Stöger, B.; Veiros, L. F.; Kirchner, K. Efficient hydroboration of alkenes and *trans*-diboration of alkynes catalyzed by Mn(I) alkyl complexes. *Angew. Chem. Int. Ed.* **2021**, *60*, 24488–24492.

(4) Garduño, J. A.; García, J. J. Non-Pincer Mn(I) Organometallics for the Selective Catalytic Hydrogenation of Nitriles to Primary Amines. *ACS Catal.* **2019**, *9*, 392-401.
